# Piezoelectric Bilayer Nickel‐Iron Layered Double Hydroxide Nanosheets with Tumor Microenvironment Responsiveness for Intensive Piezocatalytic Therapy

**DOI:** 10.1002/advs.202404146

**Published:** 2024-08-13

**Authors:** Shaohua Liu, Jianchun Bao, Boshi Tian, Shuyao Li, Meiqi Yang, Dan Yang, Xuyun Lu, Xueliang Liu, Shili Gai, Piaoping Yang

**Affiliations:** ^1^ Jiangsu Collaborative Innovation Center of Biomedical Functional Materials School of Chemistry and Materials Science Nanjing Normal University Nanjing 210023 P. R. China; ^2^ Key Laboratory of Superlight Materials and Surface Technology Ministry of Education College of Material Science and Chemical Engineering Harbin Engineering University Harbin 150001 P. R. China; ^3^ The Key Laboratory of Rare Earth Functional Materials and Applications Zhoukou Normal University Zhoukou 466001 P. R. China

**Keywords:** excitonic effect, ferroptosis, layered double hydroxide, piezocatalytic therapy, TME‐responsive

## Abstract

Piezocatalytic therapy (PCT) based on 2D layered materials has emerged as a promising non‐invasive tumor treatment modality, offering superior advantages. However, a systematic investigation of PCT, particularly the mechanisms underlying the reactive oxygen species (ROS) generation by 2D nanomaterials, is still in its infancy. Here, for the first time, biodegradable piezoelectric 2D bilayer nickel‐iron layered double hydroxide (NiFe‐LDH) nanosheets (thickness of ≈1.86 nm) are reported for enhanced PCT and ferroptosis. Under ultrasound irradiation, the piezoelectric semiconducting NiFe‐LDH exhibits a remarkable ability to generate superoxide anion radicals, due to the formation of a built‐in electric field that facilitates the separation of electrons and holes. Notably, the significant excitonic effect in the ultrathin NiFe‐LDH system enables long‐lived excited triplet excitons (lifetime of ≈5.04 µs) to effectively convert triplet O_2_ molecules into singlet oxygen. Moreover, NiFe‐LDH exhibited tumor microenvironment (TME)‐responsive peroxidase (POD)‐like and glutathione (GSH)‐depleting capabilities, further enhancing oxidative stress in tumor cells and inducing ferroptosis. To the best of knowledge, this is the first report on piezoelectric semiconducting sonosensitizers based on LDHs for PCT and ferroptosis, providing a comprehensive understanding of the piezocatalysis mechanism and valuable references for the application of LDHs and other 2D materials in cancer therapy.

## Graphical abstract

Ultrathin NiFe‐LDH nanosheets with tumor microenvironment‐responsive and GSH‐depletion ability are developed for the first time for enhanced piezocatalytic therapy and ferroptosis. The piezoelectric semiconductor showed outstanding ^1^O_2_ generation ability arising from the large excitonic effect in NiFe‐LDH. This study pioneers the development of piezocatalytic tumor therapy in two‐dimension ultrathin LDH materials, effectively inducing the generation of reactive oxygen species.

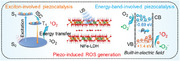

## 1 Introduction

Ultrasound (US)‐mediated tumor therapy is emerging as a valid therapeutic modality because of its noninvasive nature, ability to penetrate deep into tissues, reasonable specificity, and precise targeting.^[^ ^1^ ^]^ Like photodynamic therapy (PDT), US irradiation can generate reactive oxygen species (ROS) and trigger apoptosis in tumor cells.^[^ ^2^ ^]^ In contrast to the limited tissue penetration of light (at the millimeter scale), US exhibits superior tissue‐penetrating ability at depths exceeding 10 cm.^[^ ^3^ ^]^ Unlike the typical spontaneous Fenton reaction in chemodynamic therapy, US‐mediated therapy can induce ROS generation in response to external US stimulation, which can reduce potential side effects on normal tissues.

Recently, piezocatalytic therapy (PCT) has emerged as a novel US‐mediated therapeutic modality for the generation of ROS, such as ·OH, ·O_2_^−^, and ^1^O_2_.^[^ ^3^, ^4^ ^]^ With a similar principle of sonodynamic therapy (SDT), a sonosensitizer, i.e., piezoelectric materials, can be excited by low‐frequency ultrasonic waves for the generation of activated electrons and holes, which can participate in redox reactions with O_2_ or H_2_O to yield ·O_2_^−^ and ·OH, respectively.^[^ ^5^ ^]^ Notably, PCT has distinct advantages over SDT. In general, the rapid recombination of electrons and holes in SDT is a significant obstacle to achieving efficient ROS generation, thereby limiting therapeutic outcomes.^[^ ^1g^ ^]^ In contrast, the application of piezoelectric sonosensitizers facilitates the generation of a built‐in electric field that promotes the dissociation of electron‐hole pairs, thereby significantly amplifying the efficiency of piezocatalysis.^[^ ^6^ ^]^ Sonosensitizers inherently and efficiently influence the production of ROS owing to their essential nature. Recently, several piezoelectric sonosensitizers including ZnO, BaTiO_3_, and Bi‐based nanosheets have been developed for PCT.^[^ ^3^, ^4^, ^5^, ^7^ ^]^ For instance, our group first reported the use of 2D Bi‐based materials, including Bi_2_MoO_6_ and oxygen‐vacancy‐rich BiO_2−x_ nanosheets, as piezoelectric sonosensitizers for highly efficient tumor therapy.^[^ ^3^, ^5^ ^]^ Despite significant efforts, piezoelectric sonosensitizers remained limited. Most currently available piezoelectric sonosensitizers exhibit non‐degradability and low ROS generation capacity. Hence, the exploration of novel piezoelectric materials with highly efficient ROS generation and degradability for potential clinical applications in piezocatalytic tumor therapy has garnered significant interest.

2D nanomaterials have attracted much attention in catalysis and cancer therapy due to the complete exposure of their surface atoms, easy modification, and adjustable electrical band structure.^[^ ^8^ ^]^ The few‐atom‐thick layered structure and 2D conducting channels facilitated the rapid migration of charges to the surface of the catalysts with a reduced recombination rate.^[^ ^8^, ^9^ ^]^ Moreover, 2D materials typically exhibit significantly high exciton binding energies (> 100 meV) due to the reduced electronic screening, indicating the presence of strongly bound excitons (or electron‐hole pairs).^[^ ^10^ ^]^ Thus, pronounced excitonic effects must be considered in 2D material systems. Although a large excitonic effect can hinder charge transfer, it can facilitate the conversion of O_2_ to ^1^O_2_ through energy‐transfer processes involving the activation of spin‐triplet excitons upon excitation.^[^ ^11^ ^]^ Hence, in 2D material systems, bond excitons can facilitate the generation of ^1^O_2_ through energy transfer processes, while separated free charges can react with absorbed substrates (H_2_O or O_2_) to generate O_2_^−^ or OH, thereby significantly enhancing the ROS generation. Layered double hydroxide (LDH) nanosheets have shown promise for biomedical applications owing to their biodegradability, good biocompatibility, and unique chemical activities.^[^ ^12^ ^]^ In particular, the inherent acid‐triggered biodegradation of LDH can minimize their long‐term biological toxicity risks.^[^ ^12^, ^13^ ^]^ Notably, previous studies have demonstrated that some 2D layered bulk materials with centrosymmetric properties, such as hexagonal boron nitride (h‐BN) and transition metal dichalcogenides (TMDs), display piezoelectricity when reduced to a single or few layers due to the loss of their inversion centers.^[^ ^14^ ^]^ Inspired by this, we hypothesized that ultrathin‐layered LDH might exhibit a piezoelectric effect under external force, leading to the generation of ROS. Although certain TMDs exhibited significant piezoelectric catalytic effects for tumor therapy, the single treatment modality makes it difficult to eliminate the tumor.^[^ ^15^ ^]^ What is more, their biological safety risk and long‐term toxicity remain uncertain, which is a significant barrier to their clinical translation. Compared to TMDs, 2D LDHs showed several significant advantages. For instance, the high biocompatibility, low cytotoxicity, high cellular uptake efficiency, and inherent acid degradability can release bioactive transition metal ions (such as Fe, Cu, Mn, *etc*.) within the acidic tumor microenvironments (TME) to induce tumor apoptosis, ferroptosis, and even immune response.^[^ ^16^ ^]^ However, the use of LDH as piezoelectric sonosensitizers in piezocatalytic tumor therapy has not been reported. To date, only a limited number of studies have reported the use of MgFe and CoW‐LDH nanosheets with thicknesses exceeding 3 nm as universal sonosensitizers for SDT.^[^ ^12^, ^16^, ^17^ ^]^ Nevertheless, the current investigation of 2D LDH, particularly the mechanisms underlying ROS generation in tumor therapy, remains in its infancy. Therefore, a comprehensive study is warranted to elucidate the underlying mechanism of ROS generation in these emerging 2D LDHs, thereby facilitating the in‐depth exploration of novel 2D piezoelectric sonosensitizers for tumor therapy.

**Scheme 1:**
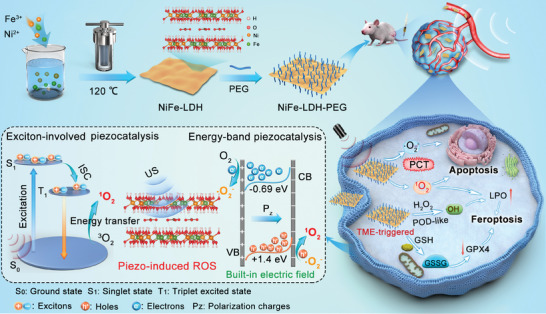
Illustration of the synthesis of bilayered ultrathin NiFe‐LDH nanosheets and their piezocatalysis mechanism for enhanced PCT and ferroptosis.

In this study, we developed ultrathin bilayer NiFe‐LDH nanosheets (thickness of ≈1.86 nm) as novel piezoelectric semiconducting sonosensitizers (*E*_g_ = 2.09 eV) with TME‐responsive enzymatic activity and glutathione (GSH)‐depleting capacity for highly efficient piezocatalytic cancer therapy and ferroptosis (**Scheme** **1**). We proposed the mechanism of US‐generated ^1^O_2_ based on the exciton effects in a 2D ultrathin LDH system. The 2D ultrathin NiFe‐LDH exhibited a remarkable piezoelectric effect with a high piezoelectric coefficient (23.17 pm V^−1^) and a pronounced excitonic effect. The opposing migration of polarized charges under US irradiation creates a built‐in electric field, which facilitates the separation of US‐induced electrons and holes, thereby enhancing the production of·O_2_^–^ by utilizing reductive electrons to reduce O_2_ through the electron transfer process. Furthermore, spin‐triplet excitons could activate the triplet O_2_ molecules to form singlet ^1^O_2_ through an energy transfer process, owing to the significant excitonic effect in 2D bilayered NiFe‐LDH systems. Our study elucidated the piezocatalysis mechanism of ultrathin NiFe‐LDH. It is expected to provide profound insights and valuable references for LDH and other 2D materials in cancer therapy. Moreover, NiFe‐LDH exhibited pH‐triggered peroxidase (POD)‐like enzymatic activity to generate toxic ·OH, thereby targeting tumor cells while minimizing the potential damage to healthy cells. Furthermore, the GSH‐depleting ability of NiFe‐LDH, resulting from the mixed‐valence Fe(II)/(III), significantly amplified the oxidative stress in tumor cells, thereby achieving highly efficient therapeutic outcomes. Benefiting from the enhanced production of ROS and depletion of GSH, NiFe‐LDH‐PEG exhibited a remarkable ability to expedite the accumulation of lipid peroxide (LPO) and deactivate glutathione peroxidase 4 (GPX4), thereby inducing ferroptosis in tumor cells. Notably, NiFe‐LDH can undergo degradation under acidic conditions, thereby facilitating its elimination from the body and minimizing its potential long‐term biotoxicity. Taken together, these results collectively demonstrate for the first time that bilayer NiFe‐LDH nanosheets with TME responsiveness and degradability have the potential as safe piezoelectric semiconducting sonosensitizers for tumor therapy, thus expanding the biomedical applications of 2D LHD materials and further extending tumor therapy.

## 2 Results and Discussion

**Figure 1:**
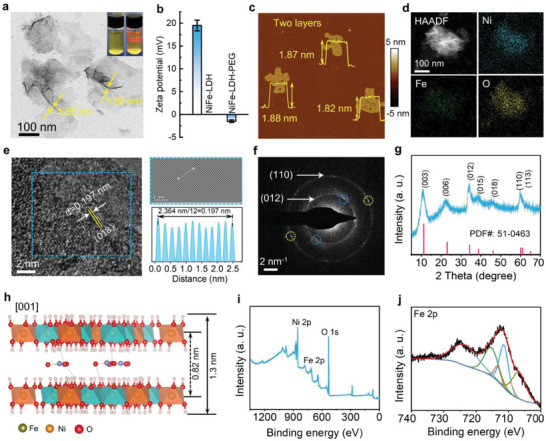
Characteristics of bilayer NiFe‐LDH nanosheets. a) TEM image of NiFe‐LDH nanosheets. b) Zeta potentials of NiFe‐LDH and NiFe‐LDH‐PEG. c) AFM image, and d) HAADF‐STEM image and corresponding element mapping of NiFe‐LDH. e) High‐resolution TEM image and interplanar spacing pattern of NiFe‐LDH. f) SAED pattern, and g) powder XRD patterns of NiFe‐LDH. h) Crystal structure diagram of NiFe‐LDH. i) XPS spectrum of NiFe‐LDH, and j) XPS spectrum of Fe 2p.

Ultrathin bilayer NiFe‐LDH nanosheets were synthesized by a facile hydrothermal approach using ethylene glycol (EG) and water as mixed solvents. Subsequently, they were modified with polyethylene glycol (PEG) to improve their water solubility and biocompatibility (Scheme 1). Fourier transform infrared (FT‐IR) spectroscopy results revealed the successful modification of NiFe‐LDH with PEG (Figure S1, Supporting Information). As displayed in Figure S2 (Supporting Information), the NiFe‐LDH‐PEG showed good dispersibility in water, PBS, and Dulbecco's modified Eagle medium (DMEM), respectively. Transmission electron microscopy (TEM) image of NiFe‐LDH (**Figure** **1**a) exhibited flexible and transparent features with an average lateral size of less than 2 nm, suggesting an ultrathin 2D nanostructure. The hydrodynamic diameter of NiFe‐LDH determined by DLS was ≈300 nm (Figure S3, Supporting Information), indicating that the appropriate size of NiFe‐LDH enabled it to accumulate in tumors by the enhanced permeability and retention（EPR) effect. Moreover, the apparent Tyndall phenomenon indicates the excellent colloidal stability of the as‐prepared NiFe‐LDH nanosheets (inset of Figure 1a), which was further confirmed by their zeta potential (+ 19.8 mV) (Figure 1b). In addition, the zeta potential decreased to –1.5 mV after PEG modification due to the inherent negative charge of PEG. Furthermore, the atomic force microscope (AFM) images in Figure S4 (Supporting Information) and Figure 1c revealed an average height distribution of ≈1.86 nm for the NiFe‐LDH nanosheets. Figure 1d shows high‐angle annular dark‐field scanning transmission electron microscopy (HAADF‐STEM) images of NiFe‐LDH. As shown, Ni, Fe, and O were uniformly distributed in the NiFe‐LDH matrix. In addition, the molar ratio of Ni to Fe was determined to be ≈3:1 by inductively coupled plasma optical emission spectrometry (ICP‐OES), consistent with the results of energy‐dispersive X‐ray (EDX) spectrometry (Figure S5, Supporting Information). As shown in Figure 1e, a distinct lattice fringe of 0.197 nm was observed, which could be assigned to the (018) plane of hexagonal NiFe‐LDH. Selected‐area electron diffraction (SAED) patterns exhibited two well‐matched rings with lattice spacings of ≈0.26 and 0.15 nm (Figure 1f), corresponding to the (012) and (110) planes of NiFe‐LDHs, respectively. The SAED pattern also revealed distinct diffraction dots, indicating the exceptional crystallinity of NiFe‐LDH. The powder X‐ray diffraction (XRD) pattern shows that the NiFe‐LDH nanosheets possess typical characteristics of hexagonal lattices with *R*3*m* rhombohedral symmetry (JCPDS No. 51–0463) (Figure 1g). Moreover, the distinct reflections of the (003) and (006) planes revealed the layered structure of NiFe‐LDH.^[^ ^18^ ^]^ The interlayer distance (*d*‐spacing) of NiFe‐LDH was calculated to be 0.82 nm (2*θ *= 10.74°) according to the Bragg equation (2*d*sin*θ *= n*λ*) (Figure 1h), further confirming the stacked layered structure of NiFe‐LDH. According to the interlayer distance (0.82 nm) and the thickness of the LDH monolayer (0.48 nm) (Figure 1h),^[^ ^18^ ^]^ the theoretical interlayer height of bilayer NiFe‐LDH was calculated to be 1.3 nm. This calculated theoretical thickness closely approximated the actual thickness of NiFe‐LDH (≈1.86 nm), revealing the bilayer ultrathin structure of NiFe‐LDH. The experimental thickness was greater than the theoretical value of the bilayer NiFe‐LDH, possibly owing to the presence of adsorbed substances.^[^ ^19^ ^]^ The X‐ray photoelectron spectroscopy (XPS) spectrum of NiFe‐LDH revealed the presence of Ni, O, and Fe (Figure 1i). Figure 1j shows a high‐resolution Fe 2p XPS spectrum. The primary peaks at 712.2 and 725.3 eV were indexed to Fe 2p_3/2_ and Fe 2p_1/2_ orbitals, respectively. The peaks at 855.3 and 872.9 eV were assigned to Ni 2p_1/2_ and Ni 2p_3/2_ orbitals, respectively (Figure S6a, Supporting Information). The O 1s XPS spectrum of NiFe‐LDH revealed three peaks at 532.6, 531.5, and 530.7 eV (Figure S6b, Supporting Information), corresponding to the chemisorbed or dissociated oxygen, oxygen vacancy region, and lattice oxygen, respectively. As shown in Figure S7 (Supporting Information), the N_2_ absorption‐desorption isotherm revealed that the NiFe‐LDH possessed a large specific Brunauer–Emmett–Teller (BET) surface area of ≈83.9 m^2^ g^−1^. This large surface area endowed NiFe‐LDH with abundant active sites for catalytic reactions.

**Figure 2:**
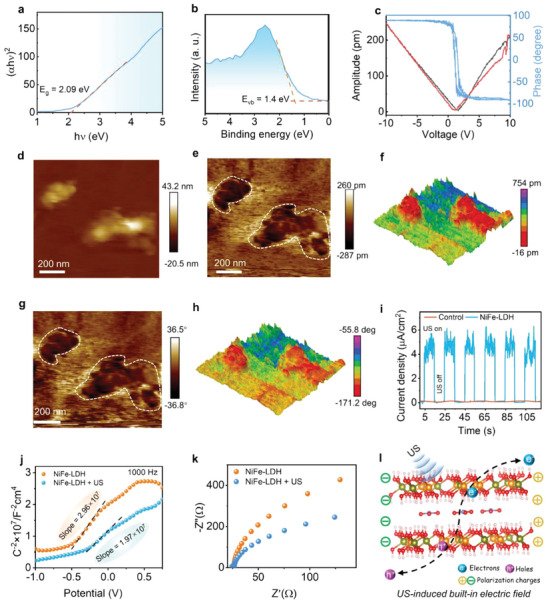
Energy band structure and piezoelectric effect of NiFe‐LDH. a) Energy bandgap, and b) XPS valence band of NiFe‐LDH. c) Amplitude–voltage and phase loops of NiFe‐LDH. d) Topography, e,f) PFM amplitude, and g,h) PFM phase images of NiFe‐LDH. i) Transient piezoelectric current of NiFe‐LDH under US irradiation (1.0 W cm^−2^, 1.0 MHz). j) Mott–Schottky plots, and k) electrochemical impedance spectroscopy (EIS) spectra of NiFe‐LDH with and without US irradiation. l) Proposed a mechanistic explanation for the enhanced separation of electrons and holes induced by US.

We then investigated the electronic structures of the NiFe‐LDH nanosheets. As displayed in Figure S8 (Supporting Information), the as‐synthesized NiFe‐LDH nanosheets exhibited broad absorption within the wavelength range of 300–600 nm. The energy bandgap (*E*_g_) of NiFe‐LDH was calculated to be ≈2.09 eV based on the Kubelck‐Munk equation (**Figure** **2**a).^[^ ^20^ ^]^ The valence band (VB) of NiFe‐LDH was estimated to be ≈+1.4 eV by XPS (Figure 2b). Therefore, the electron conduction band (CB) of NiFe‐LDH was calculated to be –0.69 eV by utilizing the equation, *E*_g_ = *E*_VB_–*E*_CB_, which was in agreement with the values estimated from Mott–Schottky plots (Figure S9, Supporting Information). The lower reduction potential, which was more negative than the standard redox potential of O_2_/·O_2_^−^ (−0.33 eV vs NHE), facilitated the reduction of O_2_ molecules to superoxide radicals (·O_2_^−^). The piezoelectric effect originates from the non‐centrosymmetric crystal structure of piezoelectricity.^[^ ^21^ ^]^ However, certain centrosymmetric 2D layered materials with centrosymmetry, such as h‐BN and TMDs, can lose their inversion centers when thinned down to a single or few layers, enabling these materials to exhibit piezoelectricity.^[^ ^14^, ^21^, ^22^ ^]^ Subsequently, we examined whether the synthesized bilayer NiFe‐LDH exhibits piezoelectric effects. In general, piezoelectric materials generate an electrical potential in response to external mechanical forces. Conversely, they may also produce mechanical stress in response to electric field stimuli.^[^ ^23^ ^]^ The piezoelectric properties of the NiFe‐LDH nanosheets were investigated using piezoresponse force microscopy (PFM), which is widely employed for the direct measurement of piezoelectricity. The PFM amplitude and phase curves of NiFe‐LDH are shown in Figure 2c. A typical butterfly amplitude curve was observed (Figure 2c). Also, a noticeable phase transition of ≈180° was observed within the localized piezoelectric hysteresis loop of NiFe‐LDH (Figure 2c). Figure 2d–h shows the morphology, amplitude, and phase images of NiFe‐LDH. The topographical mapping of NiFe‐LDH (Figure 2d) revealed a distinct nanosheet‐like morphology. Amplitude mapping of the PFM (Figure 2e,f) revealed a significant PFM response localized on the nanosheets, while exhibiting relatively diminished responses in the surrounding regions. In addition, a uniform polarity distribution was observed in phase mapping (Figure 2g,h). These findings validate the piezoelectric performance of NiFe‐LDH, possibly due to its ultrathin layer nanostructure.

To further evaluate the piezocatalytic effect, electron transfer in the NiFe‐LDH nanosheets under US irradiation was investigated using ultrasonic current measurements (Figure 2i). The NiFe‐LDH nanosheets exhibited a periodic sonocurrent response, with a maximum ultrasonic current density of ≈5 µA cm^−2^ upon switching the US on/off, indicating the generation of US‐generated carriers under US irradiation. Notably, the ability to separate electrons and holes plays a pivotal role in assessing the piezocatalytic activity of catalysts. Previous studies have demonstrated that external mechanical stress on piezoelectric materials can induce an internal electric field, thereby facilitating the dissociation of electrons and holes.^[^ ^4^, ^24^ ^]^ Thus, we propose that the high efficiency of electron‐hole separation in NiFe‐LDH under US irradiation can be primarily attributed to the built‐in electric field effect in the piezoelectric NiFe‐LDH. To validate this hypothesis, a series of electrochemical tests were carried out with and without US irradiation. As shown in Figure 2j, the Mott–Schottky slope (1.97 × 10^7^) under US irradiation was lower than that observed without US irradiation (2.96 × 10^7^), indicating the higher carrier density of NiFe‐LDH under ultrasonication.^[^ ^4^, ^25^ ^]^ Furthermore, a smaller semi‐circular arc was observed from Nyquist plots compared to those obtained without US irradiation (Figure 2k), indicating that more free charges were generated at the interface. These observations indicate that US irradiation promotes the separation of electrons and holes, thereby facilitating an increase in free charge density. Accordingly, the enhanced separation of electron‐hole pairs was proposed as a possible mechanism (Figure 2l). Upon US irradiation, the inversion symmetry of the bilayer NiFe‐LDH crystal was broken by a machine force, resulting in positive and negative polarization charges.^[^ ^26^ ^]^ The polarized charges migrated in opposite directions to generate a built‐in electric field when an external mechanical strain was applied. According to the general energy band theory, piezoelectric semiconductors can produce excited electrons and holes when subjected to mechanical strain,^[^ ^5b^ ^]^ however, the rapid recombination of electrons and holes can significantly reduce the concentration of free carriers. Fortunately, under US irradiation, the built‐in electric field induced by the US can drive electrons and holes to migrate in different directions,^[^ ^4^, ^27^ ^]^ leading to a significant improvement in the separation of these pairs. These collective findings demonstrate the potential of NiFe‐LDH as piezoelectric semiconductor with highly efficient carrier separation due to the built‐in electric field effect under US irradiation.

**Figure 3:**
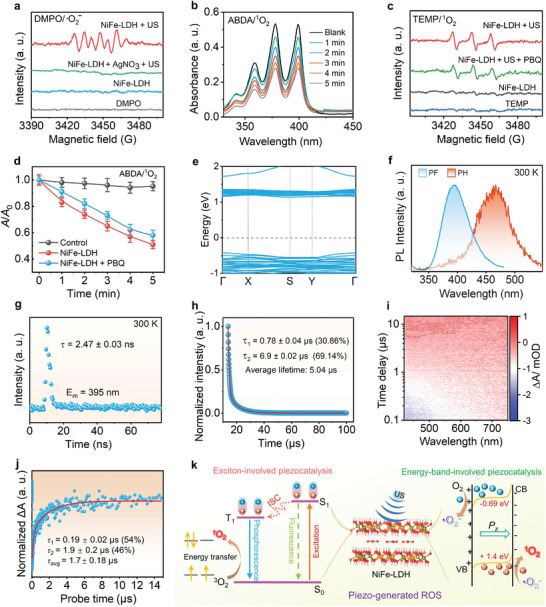
Piezoelectric catalytic performance and the mechanism of NiFe‐LDH. a) ESR spectra of ·O_2_^–^ trapped DMPO with different treatments for NiFe‐LDH. b) Time‐dependent UV–vis absorption of ABDA after treatment with NiFe‐LDH under US irradiation (1.0 MHz, 1.0 W cm^−2^). c) ESR spectra of ^1^O_2_ trapped 2,2,6,6‐tetramethylpiperidine (TEMP) with different treatments. d) Normalized UV–vis absorbance of ABDA with different treatments. Data presented as mean ± S.D. (*n* = 3). e) The calculated energy band structure of NiFe‐LDH by theoretical calculation. f) Steady‐state prompt fluorescence and phosphorescence spectra of NiFe‐LDH at 300 K (delay time 0.1 ms). g) Fluorescent decay, and h) phosphorescent decay of NiFe‐LDH at 300 K. i) TA spectra (pump: 400 nm) and j) TA kinetic traces probed at 440 nm for NiFe‐LDH. k) The piezo‐generated mechanism of ROS by NiFe‐LDH nanosheets.

Given the outstanding piezoelectric effect of the bilayer NiFe‐LDH, we next investigated its ability to generate ROS. Generally, the generation of ROS from piezoelectric semiconductors is mainly attributed to the reduction/oxidation reaction between the substrates, typically O_2_ and H_2_O, and the activated electrons and holes under US irradiation.^[^ ^4^, ^14^ ^]^ This process can induce the production of ·O_2_^–^, ^1^O_2_, and OH species. However, a suitable conduction/valence band position is required to generate ROS via the piezoelectric effect. In this study, the CB of NiFe‐LDH (–0.69 eV) was significantly more negative than ·O_2_^–^/O_2_ (–0.33 eV vs NHE), indicating that the reduction of O_2_ molecules to ·O_2_^–^ was thermodynamically favorable. Nevertheless, the lower VB (+1.4 eV) compared to H_2_O/ ·OH (+2.01 eV vs NHE) indicates the thermodynamic inhibition of ·OH generation via the oxidation of H_2_O. In particular, the generated ·O_2_^–^ species can be further oxidized to ^1^O_2_ by the holes in the valence band. To investigate the piezoelectric catalytic ability of NiFe‐LDH, 1,3‐diphenylisobenzofuran (DPBF) was used as a probe to confirm the generation of ·O_2_^−^ and ^1^O_2_.^[^ ^5a^ ^]^ As shown in Figure S10 (Supporting Information), the absorption of DPBF ≈412 nm significantly decreased after incubation with NiFe‐LDH and exposure to US irradiation, confirming the outstanding US‐induced ROS generation capacities of NiFe‐LDH nanosheets. The specific fluorescence indicator, dihydrorhodamine 123 (DHR123), was further used to confirm the production of ·O_2_^−^ and distinguish it from US‐induced ^1^O_2_.^[^ ^4f^ ^]^ A time‐dependent fluorescence intensity of DHR123 appeared for the NiFe‐LDH under US irradiation (Figure S11, Supporting Information), indicating the generation of ·O_2_^−^. Additionally, the ·O_2_^−^ generation was further verified by electron spin resonance (ESR), utilizing 5,5‐dimethyl‐1‐pyrroline‐N‐oxide (DMPO) to trap ·O_2_^−^. As depicted in **Figure** **3**a, the ESR spectra displayed a sextet peak of DMPO/·O_2_^−^ adducts when NiFe‐LDHs were irradiated with US, whereas no ESR signal was detected without US irradiation. These observations indicated that the O_2_ molecules were reduced to ·O_2_^−^ by the electrons at the CB, based on the energy band theory. To verify this, we used AgNO_3_ as an electron sacrificial agent for ESR measurements. As depicted in Figure 3a, upon US irradiation, no significant ESR signal appeared for the NiFe‐LDH after the addition of AgNO_3_, indirectly confirming the generation of ·O_2_^−^ via the electron transfer process. To further distinguish the types of ROS generated (^1^O_2_ or ·O_2_^–^) by the NiFe‐LDH under US irradiation, 9,10‐anthracenediyl‐bis(methylene)dimalonic acid (ABDA) was used to monitor the ^1^O_2_ generation.^[^ ^28^ ^]^ As shown in Figure 3b, the absorption intensity of ABDA at 378 nm decreased with the US irradiation time for NiFe‐LDH, whereas no obvious absorption intensity changes were observed for US irradiation alone (Figure S12a, Supporting Information), indicating the generation of ^1^O_2_ from NiFe‐LDH under US irradiation.

Additionally, we next used a singlet oxygen sensor green (SOSG) to further verify the generation of ^1^O_2_. As shown in Figure S13 (Supporting Information), an intense green fluorescence emission appeared upon US irradiation of NiFe‐LDH, indicating the generation of ^1^O_2_. ESR measurements were performed to further verify the ^1^O_2_. As shown in Figure 3c, a typical triplet signal appeared for the NiFe‐LDH + US group, whereas no signal was observed for the NiFe‐LDH or TEMP‐alone groups, further confirming the generation of ^1^O_2_. O_2_ can be reduced to ·O_2_^–^ by reductive electrons through electron transfer, followed by the subsequent oxidation of the generated ·O_2_^–^ to ^1^O_2_ by holes.^[^ ^29^ ^]^ In this case, ·O_2_^–^ is the transient intermediate state for the generation of ^1^O_2_. To investigate if the generation of ^1^O_2_ involves the production of ·O_2_^–^, we employed p‐benzoquinone (PBQ) as an ·O_2_^–^ sacrificial agent for ESR measurements and ABDA oxidation. As shown in Figure 3c, typical ESR signals, except for a slight reduction in intensity, were observed after the addition of PBQ, indicating that the generation of ^1^O_2_ was inhibited possibly because the intermediate ·O_2_^–^ was sacrificed by PBQ. Similarly, the oxidation of ABDA was slightly suppressed by the NiFe‐LDH under US irradiation in the presence of PBQ (Figure 3d; Figure S12b, Supporting Information), which is consistent with the ESR results. These findings imply that the generation pathway of ^1^O_2_ may not primarily originate from the conversion of ·O_2_^–^ species. It is possible that the generation of ^1^O_2_ involves an alternative pathway.

Considering the significant exciton binding energies (*E*_b_; usually hundreds of meV) of ultrathin 2D materials owing to reduced electronic screening, they can promote the energy transfer for the conversion of O_2_ to ^1^O_2_ by activating spin‐triplet excitons.^[^ ^10^, ^11^, ^30^ ^]^ Thus, the pronounced excitonic effects on 2D materials for the generation of ^1^O_2_ must be considered. The *E*_b_ of NiFe‐LDH could be evaluated by theoretical calculation by Wannier exciton model:^[^ ^31^ ^]^

(1) $$E_{b}=\frac{\mu e^{4}}{2\;\hbar^{2}\varepsilon_{\infty }^{2}}$$

where *µ* is the reduced effective mass, 1/($\frac{1}{m_{e}}+\frac{1}{m_{h}})$; and *ε*_∞_ is the high‐frequency dielectric constant. The *E*_b_ of NiFe‐LDH was calculated was determined to be 326.62 meV based on energy band results (Figure 3e). Thus, we speculated that the efficient generation of ^1^O_2_ could be attributed to the exciton effect exhibited by the few‐layered NiFe‐LDH. To verify this, photoluminescence measurements including steady‐state and time‐resolved prompt fluorescence (PF) and phosphorescence (PH) measurements were performed. PF usually originates from the radiative decay of singlet excitons, whereas PH stems from the triplet state via intersystem crossing (ISC). As shown in Figure 3f, the steady‐state PF curve of NiFe‐LDH at 300 K shows an intense emission at ≈395 nm, indicating a significant population of the singlet state. The time‐resolved PF spectra showed a lifetime of 2.47 ns (Figure 3g), indicating the rapid irradiative decay of the singlet state excitons. The nanosecond lifetimes imply that the singlet state (*S*_1_) excitons can be converted into a triplet state (*T*_1_) through ISC in the NiFe‐LDH system.^[^ ^32^ ^]^ The presence of triplet state excitons was further verified by the PH spectra at 300 K. As shown in Figure 3f, NiFe‐LDHs exhibit an obvious phosphorescent emission at 466 nm because of the retransition of triplet excitons back to the singlet ground state,^[^ ^11^ ^]^ providing clear evidence for the existence of triplet excitons at room temperature. Notably, the phosphorescence emission exhibited a redshift compared to the fluorescence emission owing to the relatively lower energy level of *T*_1_ compared to *S*_1_. Furthermore, time‐resolved PH spectra showed a biexponential decay process (*τ*_1_ = 0.78 ± 0.04 µs, 30.86%; *τ*_2_ = 6.9 ± 0.02 µs, 69.14%) with a prolonged phosphorescence average lifetimes (5.04 µs) at room temperature (Figure 3h), suggesting the existence of long‐lived triplet excitons within the NiFe‐LDH system. Moreover, femtosecond time‐resolved transient absorption (TA) spectroscopy was performed to further understand the exciton effect. As shown in Figure 3i, the TA spectra pattern of NiFe‐LDH showed a photoinduced absorption due to the excited‐state exciton absorption.^[^ ^30^ ^]^ To our surprise, a microsecond lifetime with an average of ≈1.7 µs was observed in the TA kinetic decay profile of NiFe‐LDH (Figure 3j), possibly originating from either oxygen vacancies or a metastable triplet state. A similar phenomenon was also reported in a 2D metal‐organic framework system by Zhang et al.^[^ ^32^ ^]^ Moreover, the singlet‐triplet energy gap (Δ*E*_ST_) was estimated to be 0.47 eV according to the energy separations between PF and PH. Such a moderate Δ*E*_ST_ may facilitate the ISC process for generating triplet excitons.^[^ ^33^ ^]^ Based on the above findings and analyses, an exciton‐mediated ^1^O_2_ generation mechanism in ultrathin 2D inorganic structures, which differs from that of the extensively reported inorganic sonosensitizers, was proposed.^[^ ^1^, ^12^, ^15^, ^34^ ^]^ As illustrated in Figure 3k, upon US irradiation, US‐induced electrons and holes could form singlet‐state excitons in the bilayered NiFe‐LDH 2D system. Subsequently, the generated excitons in *S*_1_ can return to the ground state (*S*_0_) accompanied by fluorescence or be converted to a lower‐energy triplet excited state through ISC. The excitons in *S*_1_ can undergo ISC to lower‐energy triplet‐excited states. Subsequently, typical medictriplet‐state O_2_ molecules are converted into singlet ^1^O_2_ via energy transfer between the long‐lived *T*_1_ and *S*_0_ states, accompanied by phosphorescence emission. In contrast, the generation mechanism of ·O_2_^–^ based on the energy band theory stems from the piezoelectric effect (Figure 3k). Similar to the photocatalysis process, electrons can be excited from the VB to the CB of NiFe‐LDH piezoelectric semiconductors under an ultrasonic wave stimulus,^[^ ^5b,c^ ^]^ and the generated excited electrons in the CB can react with O_2_ molecules to produce ·O_2_^–^. The built‐in electric field arising from the piezo‐potential can act as a driving force to promote the disassociation of electron‐hole pairs, thereby enhancing the piezocatalytic performance. The significant amount of ·O_2_^–^ species can also be partially oxidized into ^1^O_2_ by the holes in the VB. Thus, apart from the conventional viewpoint of charge carriers according to the energy band theory, the excitonic effect should be considered in 2D material systems.

**Figure 4:**
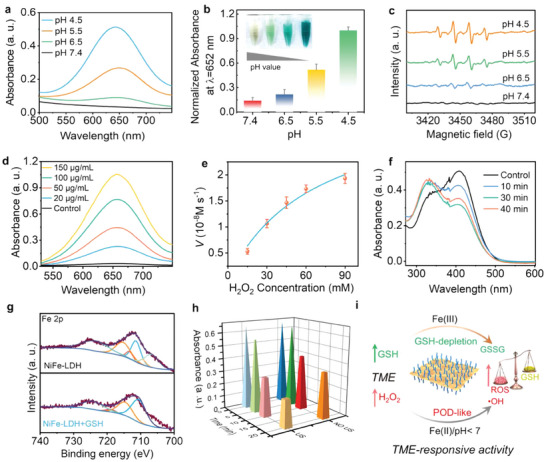
TME‐responsive enzymatic performance and GSH‐depletion of NiFe‐LDH. a,b) UV–vis absorption of TMB by NiFe‐LDH under different pH conditions (7.4, 6.5, 5.5, and 4.5). The inset shows the color change of oxidation TMB at different pH values. c) ESR spectra of NiFe‐LDH under different pH conditions. d) Concentration‐dependent UV–vis absorption spectra of oxidation TMB (oxTMB) after incubation of NiFe‐LDH at pH 5.5. e) Michaelis–Menten curves with H_2_O_2_ as a substrate. f) GSH depletion using DTNB as a probe. g) XPS spectra of Fe 2p before and after incubation with GSH. h) GSH depletion curves by NiFe‐LDH with and without US irradiation. i) Schematic illustration of the TME‐responsive enzymatic activity and GSH depletion mechanism of NiFe‐LDH.

Inspired by the remarkable POD‐like enzymatic activity exhibited by most iron‐based nanoparticles, which can convert H_2_O_2_ into ·OH,^[^ ^35^ ^]^ we investigated whether the as‐synthesized NiFe‐LDH possesses POD‐like activity. The 3,3′,5,5′‐tetramethylbenzidine (TMB) substrate was selected to evaluate the POD‐like activity of NiFe‐LDH. As shown in **Figure** **4**a, TMB transformed into blue oxTMB when exposed to NiFe‐LDH under acidic conditions, whereas no color change occurred at a pH of 7.4. This suggests that the acid‐triggered POD‐like NiFe‐LDH catalyzed the conversion of H_2_O_2_ into ·OH. Notably, oxTMB exhibits stronger absorption at 652 nm under acidic conditions (Figure 4b), suggesting that the POD‐like activity of NiFe‐LDH was enhanced by the acidic environment. Moreover, the ESR results (Figure 4c) demonstrated the generation of ·OH from NiFe‐LDH, triggered by pH variations. These findings indicate that NiFe‐LDH nanosheets exhibit intelligent generation of ·OH, specifically in the acidic conditions within the TME. In addition, both concentration and time‐dependent generation of ·OH by NiFe‐LDH was observed at a pH of 5.5 (Figure 4d; Figure S14, Supporting Information). To further evaluate the POD effect of NiFe‐LDH, a Michaelis–Menten steady‐state kinetic analysis was performed. For H_2_O_2_, the *K*_M_ and *V*_max_ values were calculated to be 156 mm and 6.25 × 10^−8^ M s^−1^, respectively (Figure 4e). In summary, NiFe‐LDH can generate significant amounts of ·OH with pH‐responsive enzymatic activity through a POD‐like catalytic reaction, thus exhibiting TME‐specific ROS generation within the TME. Overpressing endogenous glutathione (GSH) within TME can reduce tumor cell damage due to high oxidative stress, thereby suppressing therapeutic efficacy.^[^ ^36^ ^]^ Depletion of GSH is regarded as an effective strategy for augmenting oxidative stress. We then examined the ability of NiFe‐LDH to deplete GSH by utilizing 5,5′‐dithiobis (2‐nitrobenzoic acid) (DTNB) as an indicator. As shown in Figure 4f, the GSH depletion profiles indicated a significant reduction in the absorption intensity as time progressed, because the reductive GSH was oxidized to GSSG by Fe(III) in the NiFe‐LDH matrix. As shown in Figure 4g, the relative ratio of Fe(II)/Fe(III) increased from 0.98 to 1.6 after incubation of NiFe‐LDH with GSH. This indicates that certain Fe (III) ions were reduced to Fe(II) by GSH, indirectly confirming that NiFe‐LDH consumed GSH. Moreover, the NiFe‐LDH + US group exhibited a more pronounced ability to deplete GSH than the NiFe‐LDH group (Figure 4h), indicating that US irradiation enhanced the consumption of GSH by NiFe‐LDH. This explains why GSH was reduced to GSSG by the highly oxidized holes in the VB, which is consistent with our previous research findings.^[^ ^4^, ^37^ ^]^ Consequently, NiFe‐LDH could serve as TME‐responsive nanoplatforms capable of catalyzing endogenous H_2_O_2_ to generate toxic ·OH and depleting the overproduced GSH within TME (Figure 4i), thereby enhancing oxidative stress in cancer cells and inducing apoptosis.

**Figure 5:**
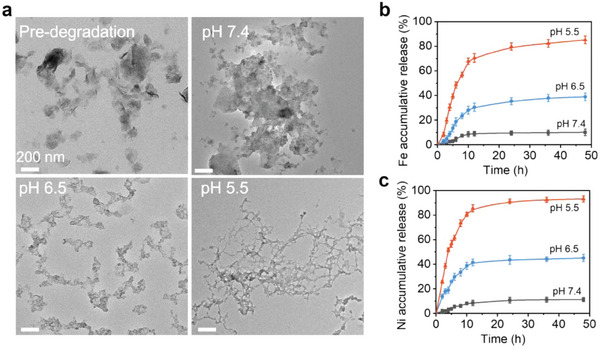
a) TEM images of NiFe‐LDH nanosheets degraded at different pH (7.4, 6.5, and 5.5) for 24 h. b) Accumulative release of Fe and c) Ni at different pH values in PBS solution. Data are shown as mean ± S.D. (*n* = 3).

Furthermore, we investigated the biodegradability of NiFe‐LDH in different pH values of PBS (pH = 7.4, 6.5, and 5.5) for 24 h. As shown in **Figure** **5**a, NiFe‐LDH gradually degraded into smaller nanosheets or nanoparticles under acidic conditions (pH 5.5), suggesting the acid‐responsive biodegradability of NiFe‐LDH. As shown in Figure 5b,c, both Ni and Fe showed pH‐triggered rapid release at the beginning and maintained a steady plateau at pH 5.5, whereas no significant Fe or Ni release was observed in a physiological environment (pH 7.4).

**Figure 6:**
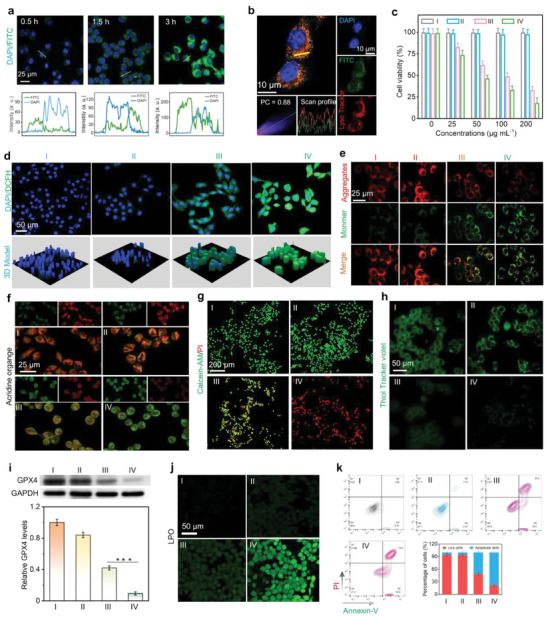
Anti‐tumor efficiency of NiFe‐LDH‐PEG nanosheets in vitro. a) CLSM images of 4T1 cell uptake after incubation with FITC‐labeled NiFe‐LDH‐PEG at various time points. Insets show the corresponding line‐scan profiles of 4T1 cells. b) Intracellular co‐localization of FITC labeled NiFe‐LDH‐PEG and lysosome in 4T1 cells. c) Cell viability of 4T1 cells with different treatments. Data presented as mean ± S.D. (*n* = 5). d) CLSM images of 4T1 cells stained with DCFH‐DA and the corresponding 3D model. e) JC‐1 with different treatments. f) CLSM images of 4T1 cells with acridine orange (AO). g) Calcein‐AM/PI double staining of 4T1 cells after different treatments. Green: live cells, Red: dead cells. h) CLSM images of 4T1 cells stained with GSH indicator (Thiol Tracker violet) with different treatments. i) Western blot analysis of GPX4 protein expression with different treatments. j) CLSM images of 4T1 cells with different treatments and stained with Liperfluo to detect lipid peroxidation (LPO). k) Flow cytometry profiles of 4T1 cells with different treatments, and the corresponding quantitative analysis. I: Control, II: US, III: NiFe‐LDH‐PEG, IV: NiFe‐LDH‐PEG + US.

Given the remarkable capability of NiFe‐LDH to generate ROS, the antitumor efficacy of NiFe‐LDH‐PEG was investigated in vitro at the cellular level. The cellular uptake of FITC‐labeled NiFe‐LDH‐PEG by 4T1 cells was investigated. Time‐dependent green fluorescence signals and the corresponding line‐scan profiles (**Figure** **6**a) verified the uptake of NiFe‐LDH‐PEG by cancer cells. The subcellular distribution of NiFe‐LDH‐PEG was determined using a co‐localization method. Notably, the green fluorescence of FITC‐labeled NiFe‐LDH‐PEG overlapped well with the red fluorescence of lysosome trackers (Lyso‐Tracker Red DND‐99) with a high Pearson correlation coefficient of 0.88 after co‐incubation for 3 h (Figure 6b). This observation revealed the entrapment of NiFe‐LDH‐PEG within the lysosomes. After incubation of normal L929 fibroblast cells with NiFe‐LDH‐PEG (0–400 µg mL^−1^), no significant cytotoxic effect was observed, indicating the good biosafety of NiFe‐LDH toward normal cells (Figure S15, Supporting Information). The in vitro piezoelectric catalytic efficiency of NiFe‐LDH‐PEG was studied using an MTT assay (Figure 6c). As shown, there was no observable cell death in the control or US groups. However, the NiFe‐LDH‐PEG group exhibited some cytotoxicity, with a cell mortality rate of 32.5% at a concentration of 200 µg mL^−1^. This tumor‐specific eradication can be attributed to its POD‐like activity, resulting from the presence of Fe(II)/Fe(III) redox couples and higher levels of endogenous H_2_O_2_ in tumor cells compared to normal cells. However, the cell mortality rate increased to 82.5% after subsequent irradiation with US, demonstrating the exceptional piezoelectric performance of NiFe‐LDH under US irradiation. Subsequently, 2′,7′‐dichlorofluorescein diacetate (DCFH‐DA) was used to evaluate the intracellular ROS levels. As shown in Figure 6d, almost no green fluorescence was observed in the control and US groups. However, the NiFe‐LDH‐PEG group exhibited a mild increase in green fluorescence, whereas the NiFe‐LDH‐PEG + US group showed the most pronounced increase in green fluorescence, indicating that most of the ROS species were generated under US irradiation.

Given the crucial function of mitochondria in controlling cell apoptosis and metabolism, the mitochondrial transmembrane potential was examined using JC‐1 as a marker to assess mitochondrial functionality. Red indicates healthy mitochondria, while green indicates injured mitochondria. As shown in Figure 6e, the cells subjected to NiFe‐LDH‐PEG treatment displayed a higher intensity of green fluorescence than the control and US groups. Notably, the NiFe‐LDH‐PEG + US group showed the most intense green fluorescence. Figure S16 (Supporting Information) shows the red/green ratio obtained by semi‐quantitative analysis based on Figure 5e. Both NiFe‐LDH‐PEG and NiFe‐LDH‐PEG + US groups exhibited lower ratios than the control and US groups. These findings indicate that the mitochondria were damaged by NiFe‐LDH‐PEG. As the endosomal membrane can be injured by ROS, we assessed its integrity by staining with acridine orange (AO). As shown in Figure 6f, the red fluorescence signal decreased after incubation with NiFe‐LDH‐PEG, implying that the integrity of the endosomal membrane was damaged by ROS. Furthermore, the red fluorescence intensity further decreased in the NiFe‐LDH‐PEG + US group, suggesting that there was an additional impairment to the integrity of the endosomal membrane, possibly due to increased ROS generation under US irradiation. Moreover, Calcein‐AM and propidium iodide (PI) co‐staining results showed that almost no green fluorescence was observed in the NiFe‐LDH‐PEG + US group (Figure 6g), indicating that most of the 4T1 cells were killed. Ferroptosis, a non‐apoptotic cell death pathway, which is featured with redox‐active iron (Fe^2+^), ROS‐induced accumulation of LPO, and inhibition of lipid peroxidation repair enzyme GPX4.^[^ ^4^, ^38^ ^]^ Considering the outstanding ROS‐generating and GSH‐depleting capacities of NiFe‐LDH‐PEG, we further examined whether the NiFe‐LDH‐PEG could induce ferroptosis in 4T1 cells. The overproduced endogenous GSH within TME can activate the expression of GPX4, which is harmful to ferroptosis. So, we first evaluated the GSH‐depleting capacity of NiFe‐LDH‐PEG in 4T1 cancer cells using Thiol Tracker Violet as a GSH probe. As shown in Figure 6h, both the control and US groups exhibited bright green fluorescence, indicating considerable levels of GSH in the 4T1 cells. Notably, the green fluorescence intensity was significantly weakened after incubation with NiFe‐LDH‐PEG, revealing the remarkable GSH depletion capacity of NiFe‐LDH‐PEG. Moreover, the green fluorescence almost disappeared after further US irradiation, indicating that US irradiation enhanced the consumption of GSH by NiFe‐LDH‐PEG. Figure S17 (Supporting Information) quantitatively shows the amount of intracellular GSH after 4T1 cells were treated with NiFe‐LDH‐PEG using GSH assay kit. As shown, the GSH levels were significantly decreased in a concentration‐dependent manner, suggesting that NiFe‐LDH‐PEG could effectively consume intracellular GSH.

Ferroptosis is characterized by the downregulation of GSH‐related GPX4 expression.^[^ ^4b^ ^]^ Figure 6i shows the GPX4 expression in 4T1 cells with different treatments. As shown, the expression of GPX4 protein in 4T1 cells was significantly down‐regulated after incubation with NiFe‐LDH‐PEG, indicating the inhibition of the repaired function of lipid antioxidants. As ferroptosis is typically accompanied by elevated levels of LPO, we next assessed the intracellular LPO levels in 4T1 cells using Lipofluo as a probe. As shown in Figure 6j and Figure S18 (Supporting Information), the cells treated with NiFe‐LDH‐PEG showed bright green fluorescence, indicating the accumulation of LPO in 4T1 cells. Notably, the brightest green signal appeared after further US irradiation compared to the other groups due to the enhanced generation of ROS by US irradiation. Collectively, these results confirm that NiFe‐LDH‐PEG can induce ferroptosis possibly owing to its GSH‐depleting and ROS generation capacities of NiFe‐LDH‐PEG. To evaluate the cell apoptosis induced by NiFe‐LDH‐PEG, a flow cytometry apoptosis assay was conducted. As shown in Figure 6k, the NiFe‐LDH‐PEG + US group showed the highest proportion of dead cells in comparison with the other groups, demonstrating the remarkable piezoelectric catalytic efficiency of NiFe‐LDH‐PEG. Collectively, these results demonstrate that biodegradable ultrathin NiFe‐LDH‐PEG nanosheets can serve as piezoelectric sonosensitizers with TME‐responsive POD‐like activity and GSH‐depleting ability to induce apoptosis of cancer cells.

**Figure 7:**
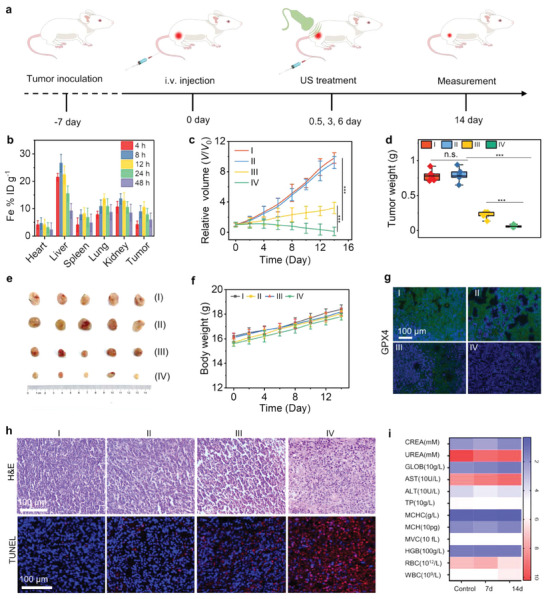
Biocompatibility and in vivo anti‐tumor efficiency of NiFe‐LDH‐PEG. a) Therapeutic procedure for mice with 4T1 tumors. b) Biodistribution of NiFe‐LDH‐PEG in major organs and tumor tissues at different time points after injection. Data presented as mean ± S.D. (*n* = 5) c) Changes in the relative volume of tumor, and d) tumor weight of mice with different treatments. Data presented as mean ± S.D. (*n* = 5) e) Representative digital images of 4T1 tumor‐bearing mice with various treatments. f) Body weight changes of mice from different groups. Data presented as mean ± S.D. (*n* = 5). g) Immunohistochemical assessment of the GPX4 expression levels in tumor tissues with different treatments. h) H&E and TUNEL staining images of the tumor with different treatments. i) Hematological indexes and biochemical parameters of mice administrated with NiFe‐LDH‐PEG. I: Control, II: US, III: NiFe‐LDH‐PEG, IV: NiFe‐LDH‐PEG + US. Data presented as mean ± S.D. (*n* = 5).

Inspired by the remarkable in vitro piezoelectric catalytic performance at the cellular level, we investigated the antitumor efficacy of NiFe‐LDH‐PEG in vivo. The in vivo antitumor effects of NiFe‐LDH‐PEG were further verified, as illustrated in **Figure** **7**a. To evaluate the tumor accumulation of the NiFe‐LDH‐PEG nanosheets, the iron content in the main organs was determined by inductively coupled plasma optical emission spectrometry (ICP‐OES). As displayed in Figure 6b, the biodistribution results demonstrated the excellent tumor uptake capacity of NiFe‐LDH‐PEG owing to the EPR of the nanoparticles and prolonged blood circulation (Figure S19, Supporting Information). The tumor‐bearing mice were randomly divided into four groups (*n* = 5): I) control, II) US, III) NiFe‐LDH‐PEG, and IV) NiFe‐LDH‐PEG + US. In the US and NiFe‐LDH‐PEG + US groups, the tumor location was irradiated with US (1.0 W cm^‒2^; 1.0 MHz) for 3 min at 12 h post‐injection. The mice were administrated and treated three times on 0.5, 3, and day 6 during 14 days of the treatment period. By monitoring the tumor volumes in each group, mice treated with PBS and US were identified to have obvious tumor growth (Figure 7c). The mice that were treated with NiFe‐LDH‐PEG had the highest rate of tumor inhibition (91.7%) of all the groups. The outstanding therapeutic efficiency indicates a superior sonodynamic efficiency in comparison to the majority of reported sonosensitizers (Table S1, Supporting Information).

However, the tumor progression was significantly restrained in the NiFe‐LDH‐PEG + US group. Digital photographs of dissected tumors in mice and changes in tumor weight confirmed the treatment effect (Figure 7d,e). Moreover, no noticeable differences in body weights were observed in any of the mice, demonstrating the absence of NiFe‐LDH‐PEG toxicity (Figure 7f). To confirm the in vivo ROS generation capacity of NiFe‐LDH‐PEG, tumor slices were collected after being treated with NiFe‐LDH‐PEG and subjected to DCFH‐DA staining for ROS detection. As shown in Figure S20 (Supporting Information), the green fluorescence signal appeared at the tumor site after the mice were treated with NiFe‐LDH‐PEG, whereas almost no green signals were observed for the control or US alone groups. This is because the NiFe‐LDH‐PEG can catalyze the H_2_O_2_ for the generation of ROS within TME through its POD‐like activity. Moreover, the green signals increased significantly for the NiFe‐LDH‐PEG + US group, indicating a high level of ROS generation after US irradiation, mainly owing to its piezocatalytic activity. Moreover, the expression of GPX4 protein was evaluated by immunostaining. As shown in Figure 7g, after the mice were treated with NiFe‐LDH‐PEG, the GPX4 expression decreased significantly compared to the control and US alone group, indicating the occurrence of ferroptosis in vivo. Moreover, the NiFe‐LDH‐PEG + US group showed the lowest GPX4 level among all groups, suggesting that US irradiation enhanced ferroptosis by promoting the generation of ROS and depletion of GSH. In addition, the therapeutic efficacy was also verified by hematoxylin and eosin (H&E) and terminal deoxynucleotidyl transferase dUTP nick‐end labeling (TUNEL) staining in each treatment group (Figure 7h). Following tumor progression, the tumors in the NiFe‐LDH‐PEG + US treatment group exhibited the broadest area of tumor necrosis in the residual tissues compared to those in the other three groups. In addition, the biocompatibility and biosafety of NiFe‐LDHs are critical during treatment. We next assessed the hemolytic effect of NiFe‐LDH‐PEG in vitro and found no significant evidence of hemolysis (Figure S21, Supporting Information). This indicates that NiFe‐LDH‐PEG has favorable blood compatibility when administered intravenously. The hematological and blood biochemical indices indicated negligible NiFe‐LDH‐PEG biological toxicity (Figure 7i). Furthermore, in each treatment group, no observable abnormalities or inflammation of major organs (heart, liver, spleen, lung, and kidney), were observed by H&E staining (Figure S22, Supporting Information). To assess the long‐term toxicity of NiFe‐LDH‐PEG, histological and blood biochemical analyses were performed on healthy BALB/c mice by intravenous injection with NiFe‐LDH‐PEG (18 mg kg^−1^). As shown in Figure S23 (Supporting Information), the blood analysis conducted on the 7 and 21st day after intravenous administration of NiFe‐LDH‐PEG did not show any significant differences compared to the control group. The blood chemistry analysis showed that the hepatic and renal function markers were within the normal range (Figure S24, Supporting Information). Moreover, the H&E staining results revealed no significant histopathological abnormalities or lesions within three weeks (Figure S25, Supporting Information), indicating negligible toxicity and excellent biocompatibility of the NiFe‐LDH‐PEG. In addition, the in vivo biodegradation and metabolism results revealed that the NiFe‐LDH‐PEG could biodegrade and excrete out of the body through fecal and renal pathways (Figure S26, Supporting Information), thereby minimizing their potential long‐term toxicity. These results collectively demonstrate that the incorporation of NiFe‐LDH‐PEG as a safe piezoelectric sonosensitizer, with the simultaneous enhancement of the POD‐like activity and GSH‐depleting ability, significantly augments the therapeutic outcomes of PCT and ferroptosis.

## 3 Conclusion

In summary, we successfully fabricated TME‐responsive bilayer piezoelectric semiconducting NiFe‐LDH nanosheets with remarkable GSH depletion and biodegradability, for enhanced PCT and ferroptosis. The obtained NiFe‐LDH could effectively generate ·O_2_^−^ through an electron transfer process under US irradiation because the built‐in electric field significantly promoted the separation of electrons and holes based on the energy band theory. Notably, upon US irradiation, NiFe‐LDH also exhibited a remarkable capacity for ^1^O_2_ generation through the energy transfer process owing to the excitonic effect in ultrathin 2D NiFe‐LDH systems. Moreover, the pH‐triggered POD‐like enzymatic activity endows NiFe‐LDH with the ability to catalyze the conversion of H_2_O_2_ to toxic ·OH within the TME under acidic conditions. Furthermore, NiFe‐LDH exhibited outstanding GSH depletion ability, further aggravating the oxidative stress in cancer cells and induce ferroptosis. In addition, NiFe‐LDH exhibited good biodegradability and biosafety, minimizing its potential long‐term biotoxicity. Therefore, the ultrathin NiFe‐LDH nanosheets can serve as a safe piezoelectric semiconducting sonosensitizer for high‐efficiency piezocatalytic tumor therapy and ferroptosis.

## 4 Experimental Section

### Synthesis and PEG Modification of NiFe‐LDH Nanosheets

NiFe‐LDH nanosheets were synthesized by a hydrothermal method. In brief, nickel (II) nitrate hexahydrate (0.6 mmol), iron (III) nitrate nonahydrate (0.15 mmol), and urea (2.0 mmol) were dissolved in a mixed solution of water (10 mL) and ethylene glycol (EG, 20 mL). After stirring at 25 °C for 30 min, the mixed solution was transferred to a Teflon‐lined stainless steel autoclave and heated at 120 °C for 12 h. The products were collected by centrifugation (10 000 rpm per min) and subsequently washed three times with water and ethanol. The power NiFe‐LDH was obtained by freeze‐drying under a vacuum. To modify the surface of NiFe‐LDH with PEG, NiFe‐LDH (50 mg) and PEG‐2000 (*M*_w_ = 2000, 20 mg) were dispersed in water (10 mL) and stirred at 25 °C for 12 h. The PEG‐modified NiFe‐LDH was then collected by centrifugation and washed three times with water.

## Conflict of Interest

The authors declare no conflict of interest.

## Supporting information

Supporting Information

## Data Availability

The data that support the findings of this study are available from the corresponding author upon reasonable request.
