# Supplementary material for: Piezoelectric Bilayer Nickel‐Iron Layered Double Hydroxide Nanosheets with Tumor Microenvironment Responsiveness for Intensive Piezocatalytic Therapy
Source: Adv Sci (Weinh). 2024 Aug 13;11(39):2404146. doi: 10.1002/advs.202404146 (PMC11497024; doi:10.1002/advs.202404146)
Supplement: Supplementary file 1 — Supporting Information [file ADVS-11-2404146-s001.docx]

Supporting Information

Piezoelectric Bilayer Nickel-Iron Layered Double Hydroxide Nanosheets with Tumor Microenvironment Responsiveness for Intensive Piezocatalytic Therapy

*Shaohua Liu, Jianchun Bao, Boshi Tian,* Shuyao Li, Meiqi Yang, Dan Yang, Xuyun Lu, Xueliang Liu, Shili Gai,* and Piaoping Yang**

**Experimental Section**

*Chemicals:* Nickel (II) nitrate hexahydrate, iron (III) nitrate, glycol (EG), dihydroorhodamine 123 (DHR123), 5,5'-dithiobis-2-nitrobenzoic acid (DTNB), 5,5-dimethyl-1-pyrroline-N-oxide (DMPO), triacetonamine hydrochloride (TEMP), 3,3′,5,5′-tetramethyl-benzidine (TMB), were purchased from Aladdin Chemical Reagent Co., Ltd. 2,7-dichlorofluorescein diacetate (DCFH-DA), 5,5,6,6′-tetrachloro-1,1′,3,3′ tetraethylbenzimi-dazoylcarbocyanine iodide (JC-1) staining kit, Calcein-AM, propidium iodide (PI), and annexin V-FITC/PI apoptosis detection kit was purchased from Beyotime Inst. Biotech. All chemicals were used as received without purification. 4T1 cells were obtained from FDCC (Ruilu in Shanghai, China).

*Characterization:* The morphology of the as-prepared NiFe LDH nanosheets was examined *via* TEM (FEI Tecnai G2 S-Twin) and atomic force microscope (AFM) (Bruker Dension Icon.). The phase purity and crystal structure of NiFe-LDH were measured *via* XRD (RigakuD/max-TTR-III). The chemical composition was determined using XPS (ThermoFisher ESCALAB 250Xi). A zeta potential instrument (Malvern Zetasizer Nan Nano ZS90) and UV-vis absorption spectra were obtained on a Shimadzu-UV2450 spectrophotometer. The photoelectrochemical performance was obtained using an electrochemical analyzer (CHI660E). UV-vis diffuse reflectance spectra (UV-vis DRS) were obtained using a UV-vis spectrophotometer (UV-2600 Shimadzu Corporation). Electron-spin-resonance (ESR) spectra were acquired by a Bruker microESR spectrometer. The dynamic light scattering measurements for different samples were performed on a Malvern Zetasizer Nan Nano ZS90. The BET surface area of various samples was determined by the Micromeritics ASAP1020 HD88 instrument. Confocal laser scanning microscope (CLSM) images were captured using a confocal microscope (Leica TCS SP8). Flow cytometry assays were conducted using a flow cytometer (BD Accuri C6). The concentrations of Fe were quantified using inductively coupled plasma optical emission spectrometry (ICP-OES). Electrochemical tests were conducted on an electrochemical analyzer (CHI660E) using Ag/AgCl as a reference electrode in the Na_2_SO_4_ aqueous solution (0.1 M). The photoluminescence and phosphorescence spectra were measured at room temperature on a fluorescence spectrophotometer (FLS980, Edinburgh Instruments). Ultrafast transient absorption (TA) spectroscopy experiments were performed under ambient conditions on a Helios pump-probe system (Ultrafast Systems).

*Theoretical Calculation*: Optimization of the structure of NiFe-LDH with was performed by using the Vienna ab initio simulation package with the projector augmented wave (PAW) pseudo potentials. A spin-polarized Perdew-Burke-Ernzerhof (PBE) parameterization of the generalized gradient approximation (GGA) was adopted for the exchange-correlation. The NiFe-LDH structure was optimized by using 400 eV as the cutoff energy for basis function. A Hubbard-U correction (DFT + U method) was applied, as implemented in the PAW, to improve the description of NiFe-LDH. The value of U is 4.3 for Fe, and 3.8 for Ni. These U values are selected according to the literature.^[1]^ The Brillouin zone integrations were performed using a 2×3×1 gamma grid for the slab models. A vacuum of 15 Å was adopted along *z*-axis. During structure optimization and electronic properties, all energy change criterion was set to 10^-5^ and 10^-6^, respectively. The atoms were relaxed until the force action on each atom was less than 0.02 eV/Å. The effective masses were obtained by using finite difference method as implemented in Effective Mass Calculator (EMC).^[2]^ The Wannier-Mott exciton binding energy was estimated by using a modified hydrogen-atom-like Bohr model.^[3]^ *E*_b_ is defined as $\text{E}_{b}=\text{ }\text{μ}\text{e}^{\text{4}}\text{/2}\text{ħ}^{\text{2}}\text{ε}_{\text{∞}}^{\text{2}}$, where $\varepsilon_{\infty}$ and $\mu$ are the high frequency dielectric constant and reduced effective mass, respectively, $\text{μ}\text{= }\text{m}_{\text{e}}^{\text{*}}\text{m}_{\text{h}}^{\text{*}}\text{/}{\text{(}\text{m}}_{\text{e}}^{\text{*}}\text{ }\text{+}{\text{ }\text{m}}_{\text{h}}^{\text{*}}\text{)}$. In this study, the effective mass of electron and hole was calculated to be 2.84 and 3.87 *m*_0_, respectively; the band energy was calculated to be 1.57 eV.

*Electrochemical Test:* The electrochemical tests were measured on an electrochemical analyzer (CHI660E) in the Na2SO4 aqueous solution (0.1 M). To prepare the working electrode, NiFe-LDH was coated on the ITO glass using Nafion/ethanol solution. Sample-coated ITO glass, the Ag/AgCl standard electrode, and the Pt wire served as working electrodes, reference electrodes, andcounter electrodes, respectively.

*Extracellular ROS Detection:* For ^1^O_2_ detection, NiFe-LDH solution (2 mL, 100 μg mL^–1^) was incubated with ABDA solution (30 µM) and irradiated with ultrasound (1 MHz, 1.0 W cm^–2^) at different time points. At given time points, the supernatant was collected to be determined by UV-vis spectrometer at peak 378 nm. For ·O_2_^–^ detection, NiFe-LDH solution (3 mL, 100 μg mL^–1^) containing DHR 123 (20 µM) was irradiated with ultrasound (1.0 W cm^–2^) for different times to record the changes of fluorescence intensity.

*POD-like Activity of NiFe-LDH:* POD-like activity assays of NiFe-LDH were performed using TMB as substrate in the presence of H_2_O_2_ in PBS buffer solution. The UV-vis absorption of TMB was recorded at different pH values.

*GSH Depletion:* NiFe-LDH (100 μg mL^–1^) was dispersed in GSH (3 mM) solution and subsequently irradiated with ultrasound for different times. After the addition of DTNB (0.1 mM), the absorbance changes of the solution were recorded using a UV-vis spectrophotometer.

*In Vitro Cellular Uptake:* 4T1 cells were seeded on a 6-well plate (10^5^ cells per well) and incubated overnight. Then, FITC-labeled NiFe-LDH-PEG (100 μg mL^–1^) was incubated with 4T1cells for 0.5, 1.5, and 3 h. Next, the cells were sequently stained with commercial Lyso-Tracker and DAPI. After rinsing the cells with PBS, the confocal laser scanning microscope was utilized to capture fluorescence images.

*In Vitro Cytotoxicity:* The cytotoxicity was carried out on 4T1 and L929 cells using the MTT assay, respectively. 4T1 or L929 cells were seeded in a 96-well plate and allowed to adhere overnight. Next, 100 μL of a solution containing different concentrations (0, 20, 50, 100, and 200 μg mL^–1^) of NiFe-LDH-PEG was introduced into each well and incubated for 24 h. The assessment of cell viability was conducted by measuring the absorbance at a wavelength of 490 nm.

*Cellular GSH Level Assay:* 4T1 cells were seeded into a cell culture dish (10^5^ cells) overnight. Next, the cells were treated with US, NiFe-LDH-PEG, and NiFe-LDH-PEG +US (100 μg mL^‒1^) for 4 h at 37 ^o^C. Then, the cellular GSH level was determined by a commercial Thiol TrackerTM Violet probe following the standard protocol.

*Intracellular ROS Assay:* In brief, 4T1 cells (10^5^ per well) were cultured in a 6-well plate for 12 h. The cells were exposed to NiFe-LDH-PEG (100 μg mL^−1^) and incubated for 4 h. The US and NiFe-LDH-PEG groups were then treated with US (1.0 MHz, 1.0 W cm^–2^) for 3 min. Afterward, DCFH-DA (10 μM) was introduced to the cells and incubated for a further 20 minutes.

*Assessment of Mitochondria Membrane Integrity:* For mitochondria integrity assay, 4T1 cells (10^5^ per well) were plated in 6-well plates and cultured overnight. After that, 4T1 cells were treated with control, US, NiFe-LDH-PEG, and NiFe-LDH-PEG + US groups. For US and NiFe-LDH groups, the cells were irradiated with the US (1.0 MHz, 1.0 W cm^–2^) for 1 min. After that, the cells were incubated with the JC-1 for 20 min and imaged on a CLSM.

*Intracellular LPO Assay*: 4T1 cells were seeded and incubated in a 6-well cell culture plate for 24 h. After that, 4T1 cells were treated with control, US, NiFe-LDH-PEG, and NiFe-LDH-PEG + US groups, respectively. The cells were washed with PBS three times. Then, 4T1 cells were stained with a lipid peroxidation probe Liperfluo (DOJINDO, L248) for 20 min. Finally, the fluorescent images were captured using a fluorescence microscope.

*Lysosomal Membrane Integrity Assay*: 4T1 cells were seeded into a 6-well plate and cultured overnight. After that, 4T1 cells were treated with control, US, NiFe-LDH-PEG, and NiFe-LDH-PEG + US groups, respectively. After the cells were washed with PBS three times, an AO solution (10 μM) was added and further incubated at 37 °C for 20 min. Finally, the images were captured using a fluorescence microscope.

*Intracellular Antitumor Activity:* 4T1 cells were initially plated in 96-well plates and incubated for 12 h. Subsequently, the cells were exposed to NiFe-LDH-PEG (100 μg mL^–1^) and incubated at a temperature of 37 °C for 4 h. After 4T1 cells were irradiated with ultrasound (1 MHz, 1.0 W cm^–2^) for 1 min, the cells were incubated for an additional 24 h at 37 °C. Last, the cell viability was evaluated by a standard MTT assay. For the live/dead cell staining assay, various treatments were applied to the cells. Afterward, Calcein-AM/PI co-staining was performed for 30 min. In the flow cytometry analysis, 4T1 cells were initially cultured in a 12-well dish for adhesion over 12 h. Subsequently, different treatments including control, US, NiFe-LDH-PEG, and NiFe-LDH -PEG + US were administered. For the NiFe-LDH and NiFe-LDH + US groups, a concentration of 100 μg mL^–1^ of NiFe-LDH-PEG was added followed by an additional culture time of 6 h. In the case of the US and NiFe-LDH-PEG + US groups, ultrasound irradiation at a frequency of 1.0 MHz with an intensity of 1.0 W cm^–2^ was applied for one minute. Finally, an annexin V-FITC/PI dual-staining apoptosis detection kit was used to treat the cells.

*Animals and Tumor Model:*Female BALB/c mice (4–6 weeks old) were obtained from Beijing Vital River Laboratory Animal Technology Co., Ltd. To create a tumor model, mice were injected with 4T1 cells (2 ×10^6^) in the right axillary fossa. *In vivo* experiments were conducted using mice bearing 4T1 tumors once the tumor volume reached about 60 mm^3^.

*In Vivo Biodistribution:* BLAB/c mice bearing 4T1 tumors (*n* = 3) were administered NiFe-LDH-PEG intravenously at a dosage of 18 mg kg^−1^. Following specific time intervals of injection including 4, 8, 12, 24, and 48 h respectively, the mice were euthanized and the primary organs such as the heart, liver, spleen, lung, and kidney were harvested for evaluating biodistribution. The Fe and Ni percentage in major organs and tumors by ICP-OES analysis.

*In Vivo Anticancer Performance:* Twenty 4T1 tumor-bearing female BALB/c mice were randomly divided into four groups (*n* = 5 per group) as follows: I) control, II) US, III) NiFe-LDH-PEG, and IV) NiFe-LDH-PEG + US. At 12 h post-injection (18 mg kg^–1^), the tumor sites were exposed to ultrasound (1 MHz, 1.0 W cm^–2^) for 3 min. The mice were administrated and treated three times on days 0, 3, and day 6 during 14 days of the treatment period. The volume of the tumor (mm^3^) was calculated using the formula V = *lw*^2^/2, where *w* and *l* represent the width and length of the tumor respectively. After 14 days following various treatments, the mice with tumors were euthanized. Main organs such as the heart, liver, spleen, lung, and kidney along with tumors were collected and subjected to staining techniques including hematoxylin and eosin (H&E) as well as terminal deoxynucleotidyl transferase dUTP nick end labeling (TUNEL).

*Statistical Analysis:* Quantitative data were presented as mean ± standard deviation (mean ± S.D.). Analysis of variance (ANOVA) was used for multiple comparisons, and the student’s *t*-test was used for two-group comparisons. Statistical significance was assumed at a value of **p* < 0.05, ***p* < 0.01, ****p* <0.001, n.s. stands for not significant. The sample size (*n*) for *in vitro* cell and *in vivo* animal study was *n* = 5.

**Figure S1.** FT-IR spectra of NiFe-LDH, NiFe-LDH-PEG, and PEG.

**Figure S2.** Photographs of aqueous suspensions of NiFe-LDH-PEG dispersed in water, PBS, and DMEM, respectively.

**Figure S3.** Hydrodynamic diameter of NiFe-LDH.

**
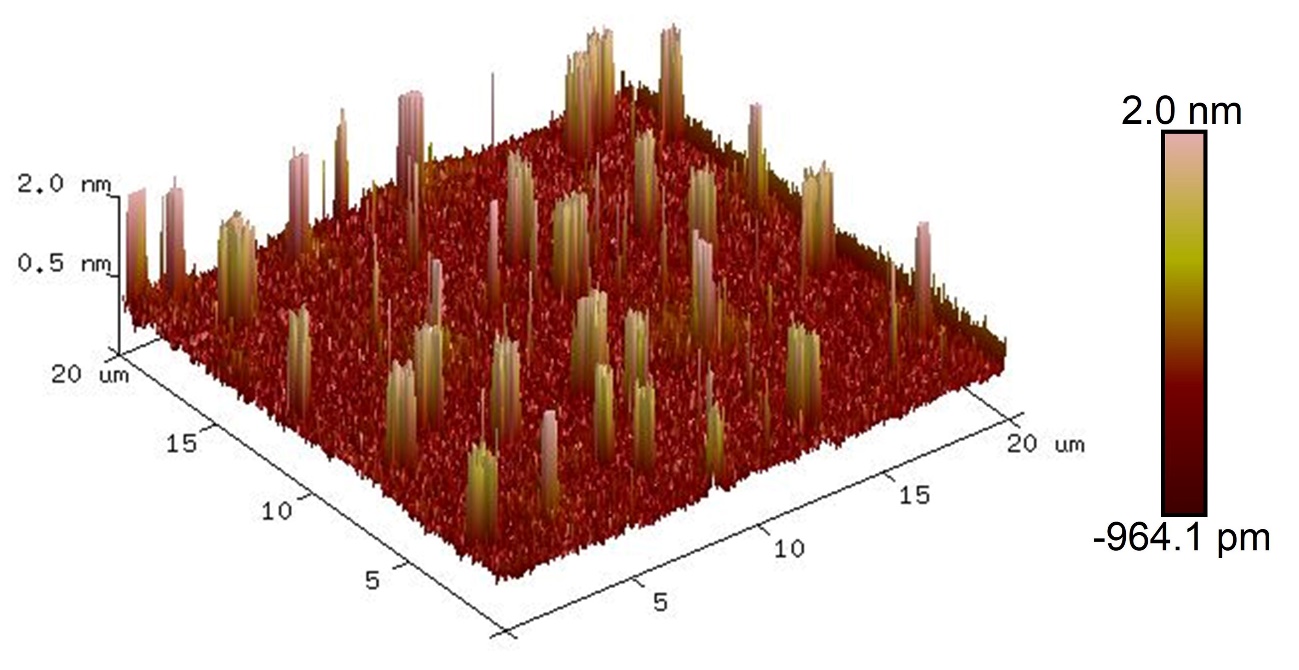
**

**Figure S4.** AFM image of NiFe-LDH.

**Figure S5.** EDS pattern of NiFe-LDH.


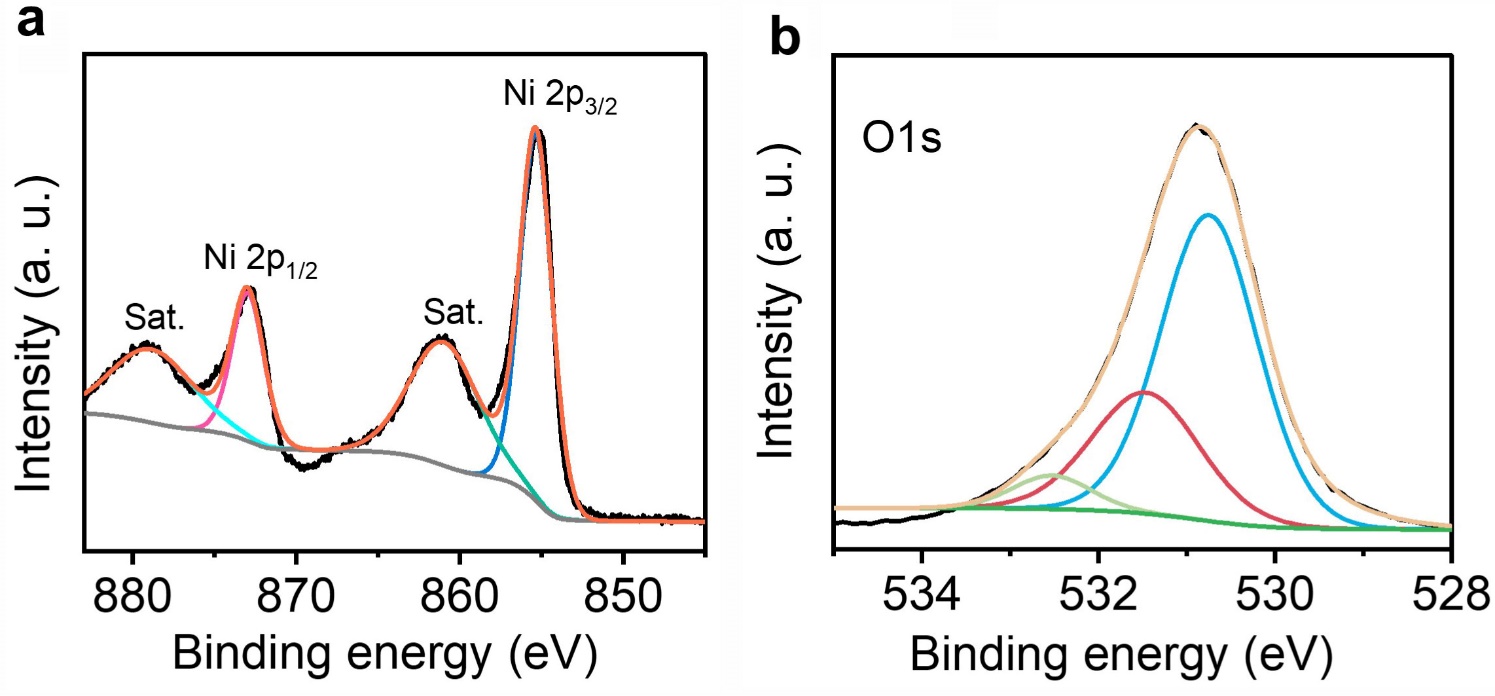


**Figure S6.** XPS spectra of a) Ni 2p and b) O1s.

**Figure S7.** N_2_ adsorption-desorption isotherm of NiFe-LDH.

**Figure S8.** UV-vis DRS spectrum of NiFe-LDH.

**Figure S9.** Mott-Schottky plots of NiFe-LDH at different frequencies.

**
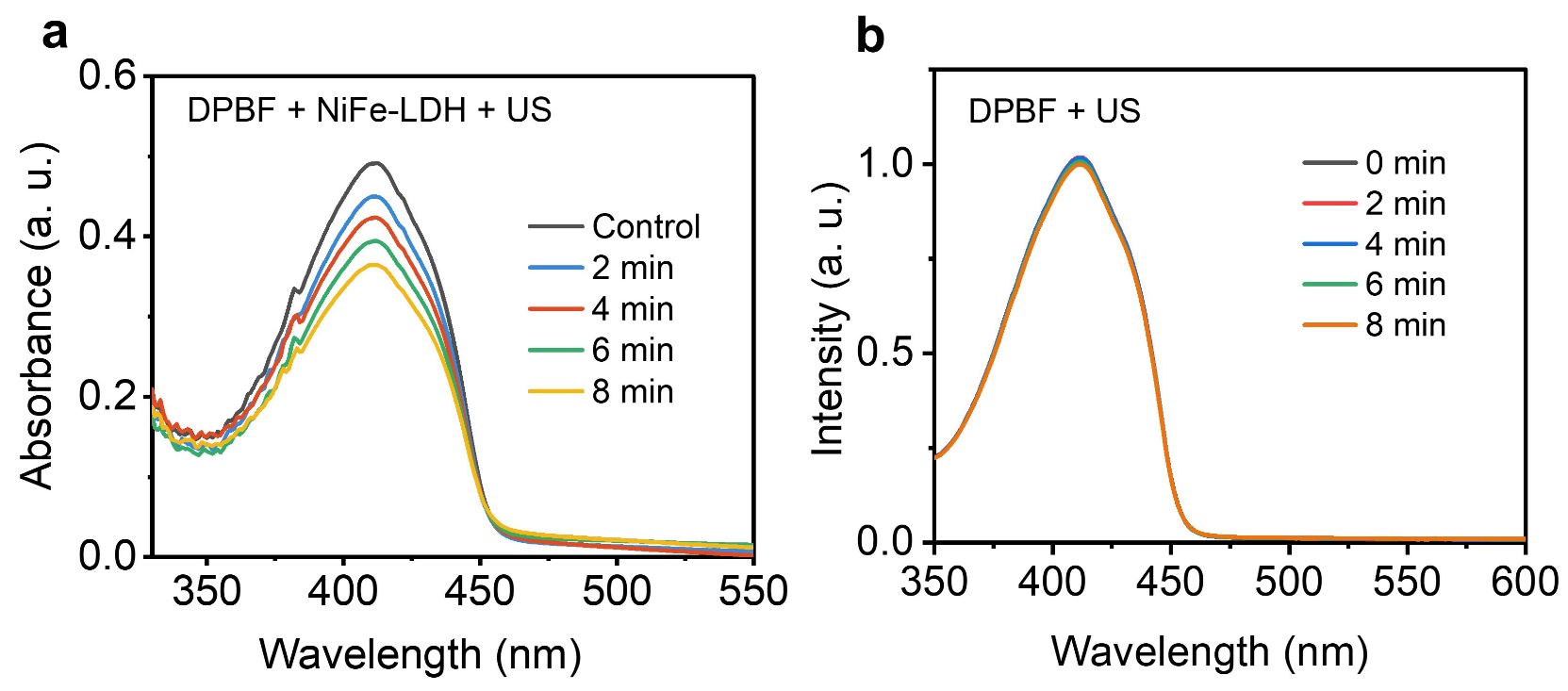
**

**Figure S10.** a) Time-dependence of UV–vis absorbance of DPBF after incubation with NiFe-LDH under US irradiation. b) Time-dependence of UV–vis absorbance of DPBF with US irradiation.

**Figure S11.** Time-dependence of fluorescence of DHR123 after incubation with NiFe-LDH under US irradiation.

**
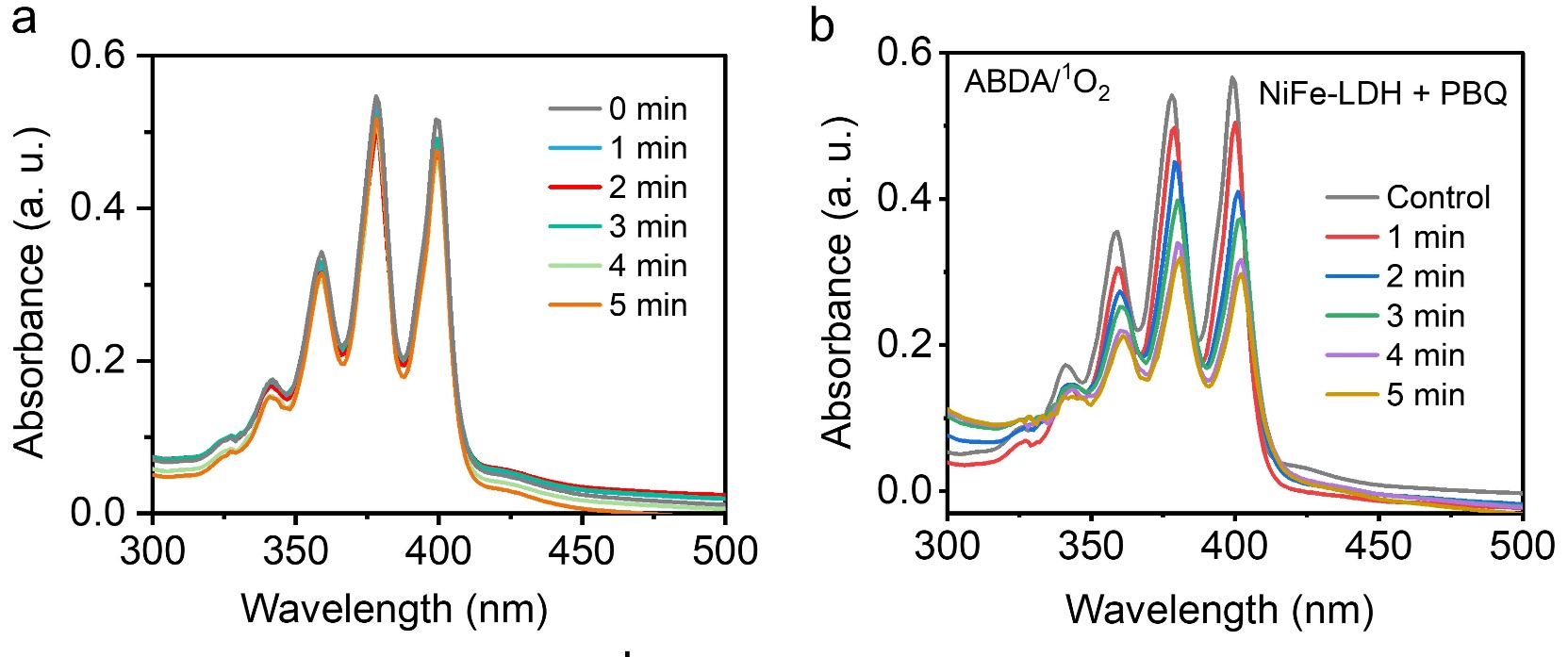
**

**Figure S12.** a) Time-dependent UV-vis absorption of ABDA under US irradiation. b) Time-dependent UV-vis absorption of ABDA after treatment with NiFe-LDH under US irradiation.

**Figure S13.** a) Time-dependent fluorescence of SOSG after treatment with NiFe-LDH under US irradiation. b) Illustration of the singlet oxygen (^1^O_2_) detections using SOSG.

**Figure S14.** Time-dependent UV-vis absorption of oxTMB after incubation with NiFe-LDH in the presence of H_2_O_2_ at pH 5.5.

**Figure S15.** Cell viability of L929 cells after incubation with NiFe-LDH-PEG for 24 h. Data are shown as mean ± S.D. (*n* = 5)

**Figure S16.** Quantitative analysis of JC-1 according to Figure 6e. Data are shown as mean ± S.D. (*n* = 3)

**Figure S17.** GSH levels in 4T1 cells at various concentrations of NiFe-LDH-PEG. Data are shown as mean ± S.D. (*n* = 3)

**Figure S18.** Relative fluorescence intensity of 4T1 cells with different treatments and stained with Liperfluo according to Figure 6j. Data are shown as mean ± S.D. (*n* = 3)

**Figure S19.** Blood circulation curve of NiFe-LDH-PEG. Data are shown as mean ± S.D. (*n* = 5)

**
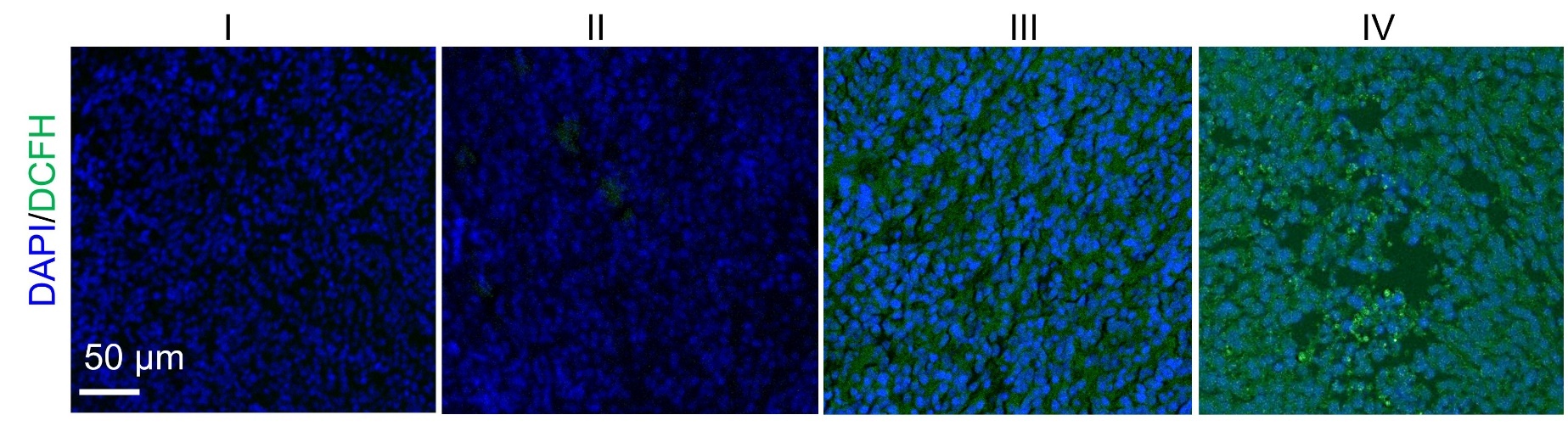
**

**Figure S20.** Fluorescence images of tumor slices stained with the ROS probe of DCFH-DA.

**
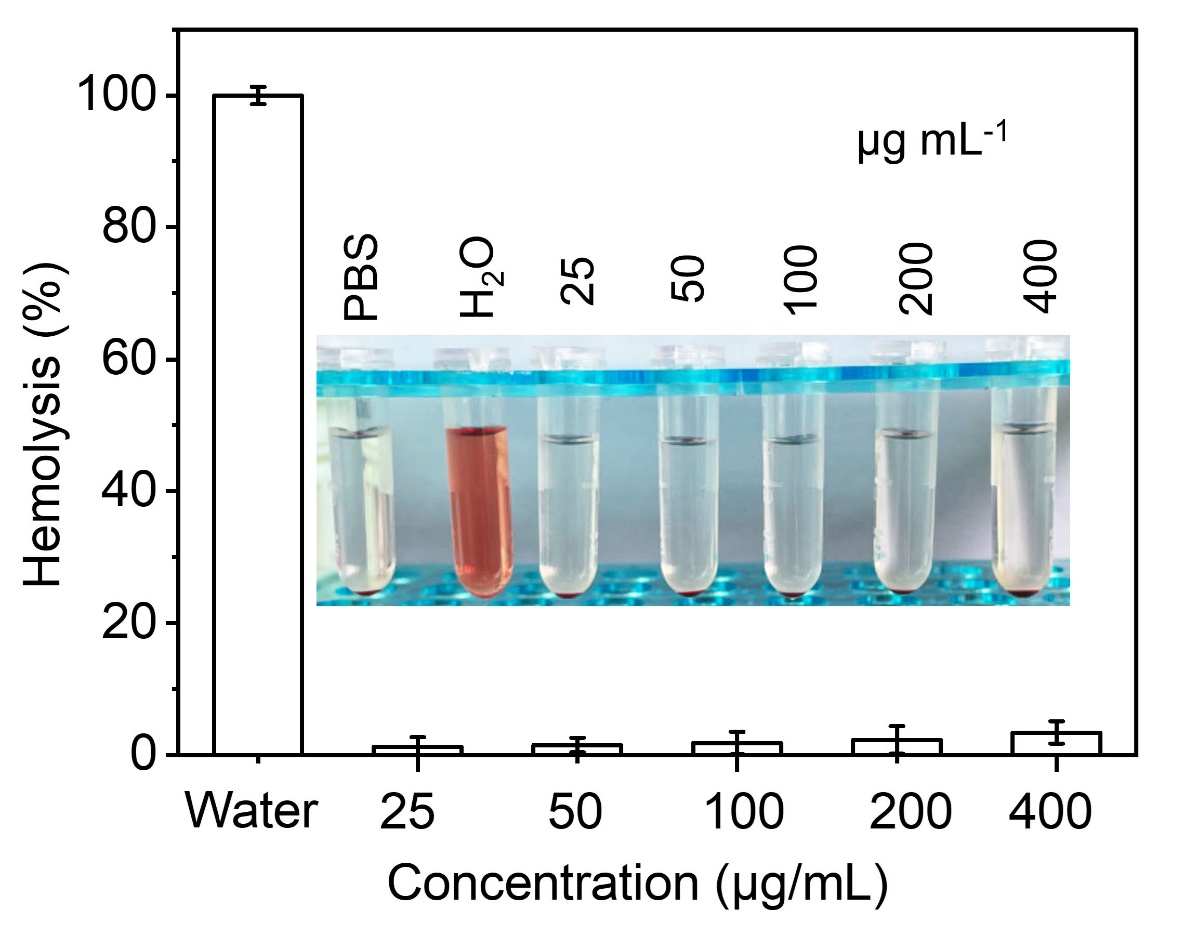
**

**Figure S21.** Hemolysis analysis of NiFe-LDH-PEG. Data presented as mean ± S.D. (*n* = 3)

**
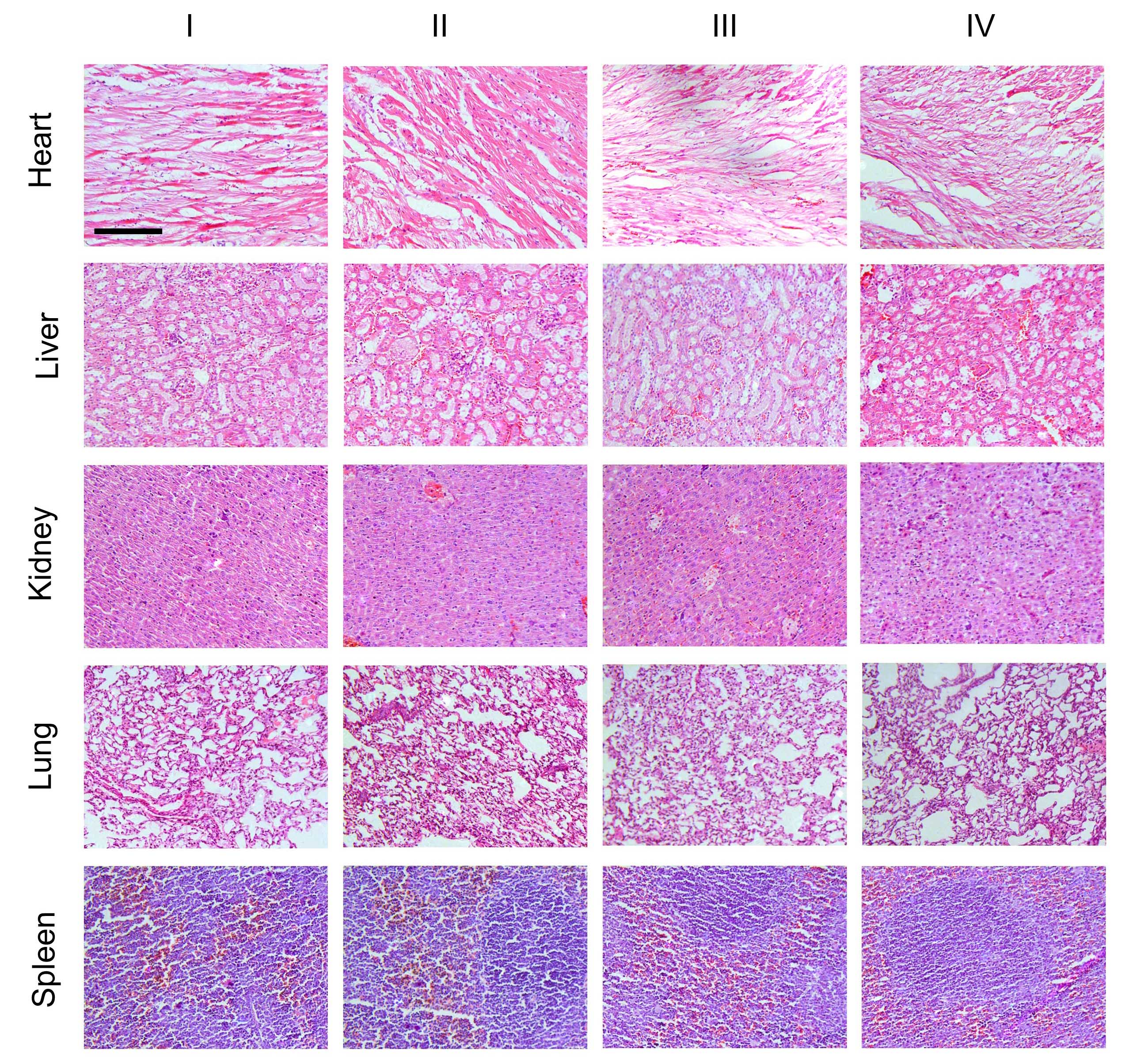
**

**Figure S22.** H&E stained photographs of the heart, liver, spleen, lung, and kidney were obtained from different groups after 14 days of treatment. I: control, II) US, III) NiFe-LDH-PEG, and IV) NiFe-LDH-PEG + US. Scale bar: 100 µm.


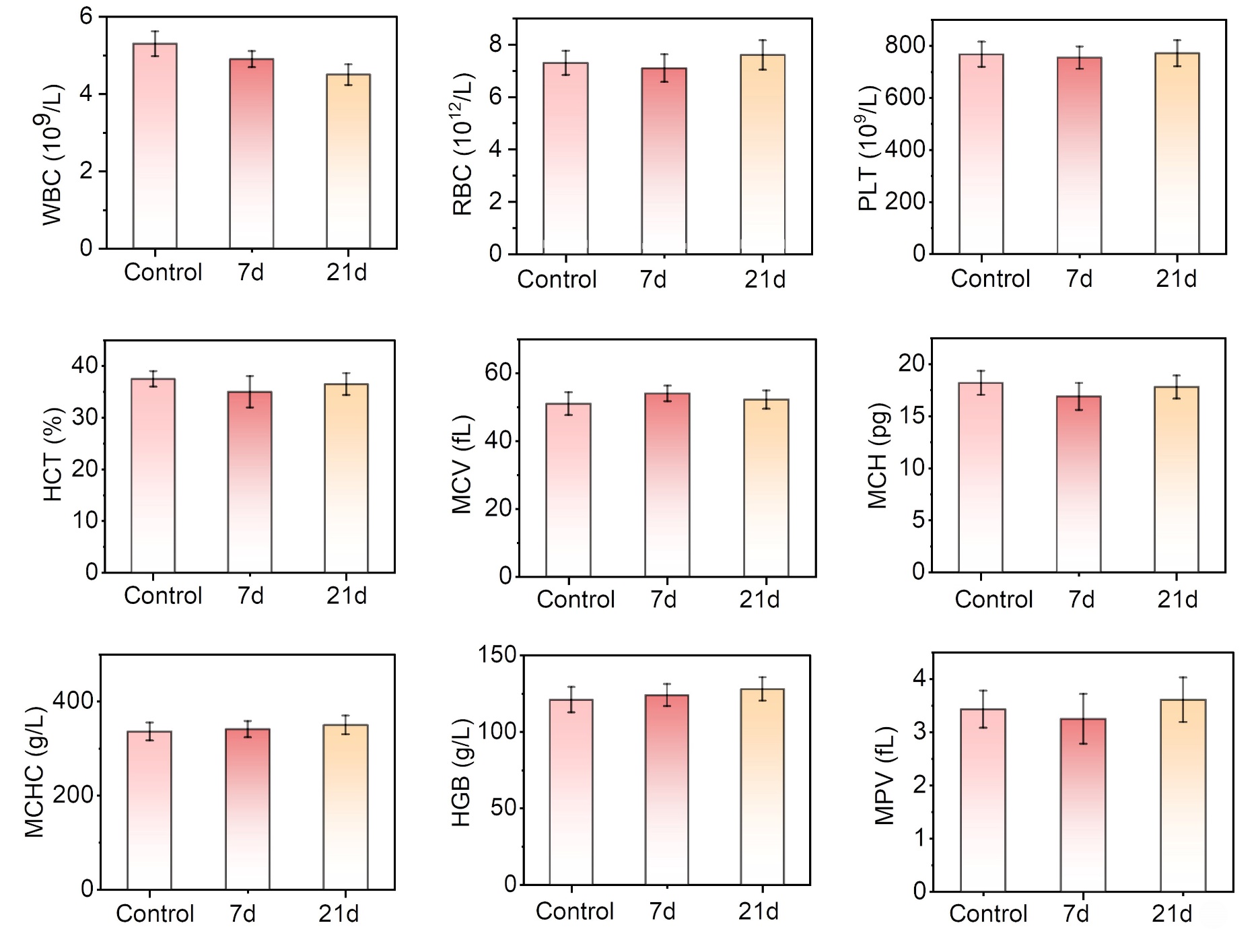


**Figure S23**. Complete blood panel analysis data of healthy mice after i.v. injection of NiFe-LDH-PEG (18 mg kg^−1^) (*n* = 3). Blood routine indexes including white blood cells (WBC), red blood cells (RBC), platelets (PLT), hematocrit (HCT), mean corpuscular volume (MCV), mean corpuscular haemoglobin (MCH), mean corpuscular haemoglobin concentration (MCHC), haemoglobin (HGB), and mean platelet volume (MPV). No significant differences occurred in all indexes.


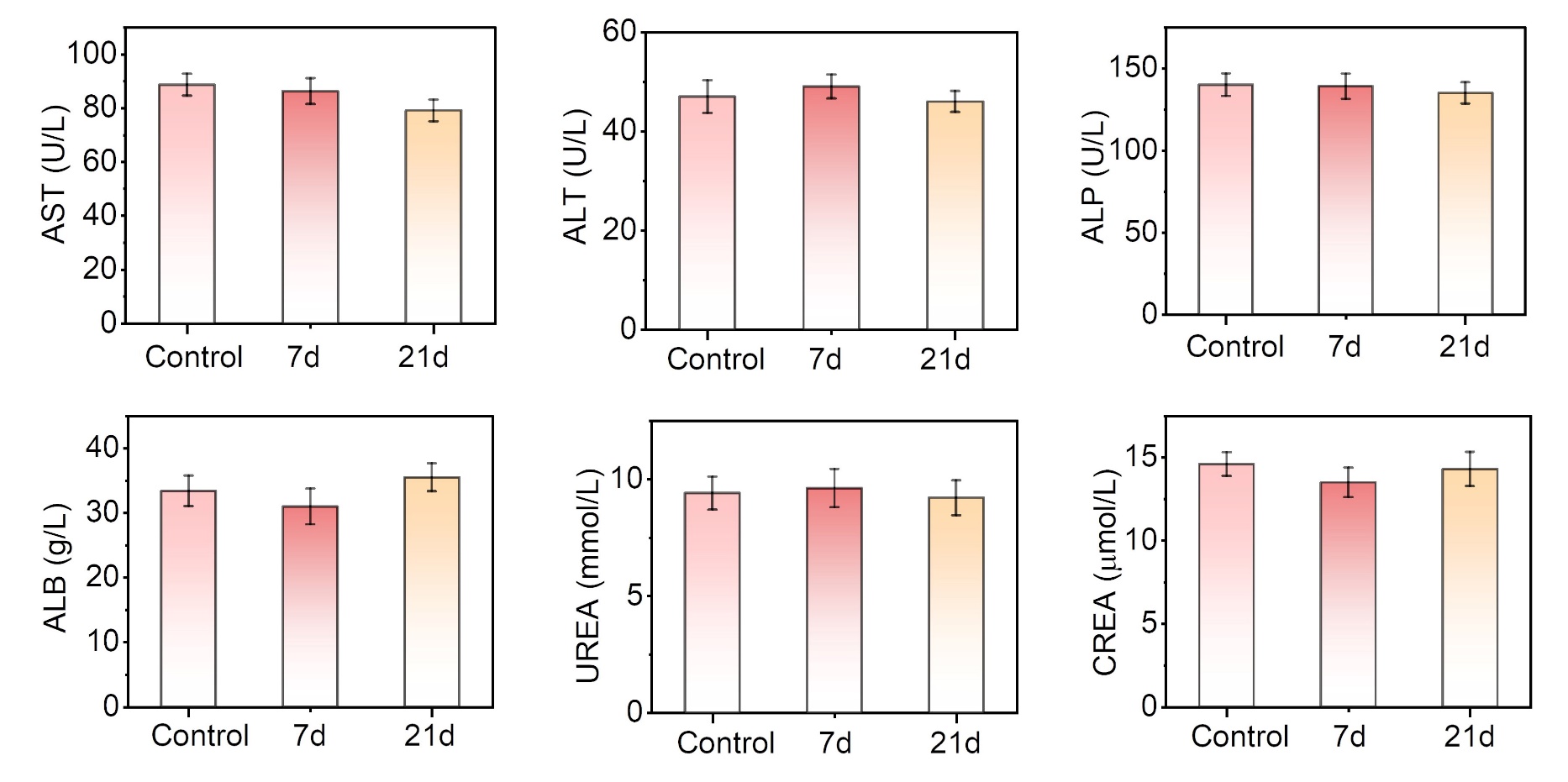


**Figure S24**. Blood biochemistry data of healthy mice after i.v. injection of NiFe-LDH-PEG (18 mg kg^−1^) (*n* = 3). Liver and renal function indexes including alanine aminotransferase (ALT), alkaline phosphatase (ALP), asparagine aminotransferase (AST), albumin (ALB), urea nitrogen (UREA), and creatinine (CREA).


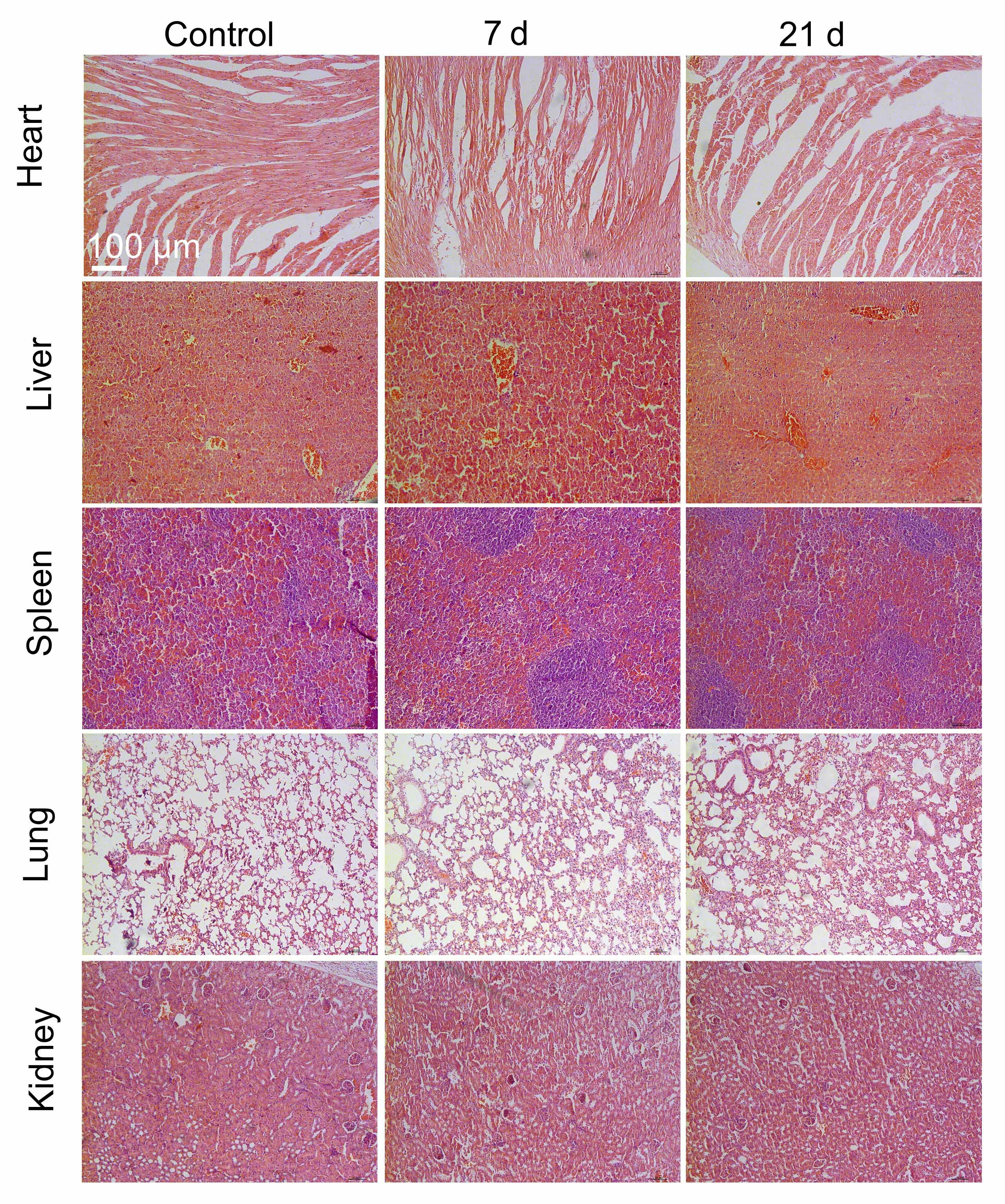


**Figure S25.** H&E staining of major organs from healthy BALB/c mice after i.v. injection of NiFe-LDH-PEG (18 mg kg^−1^).


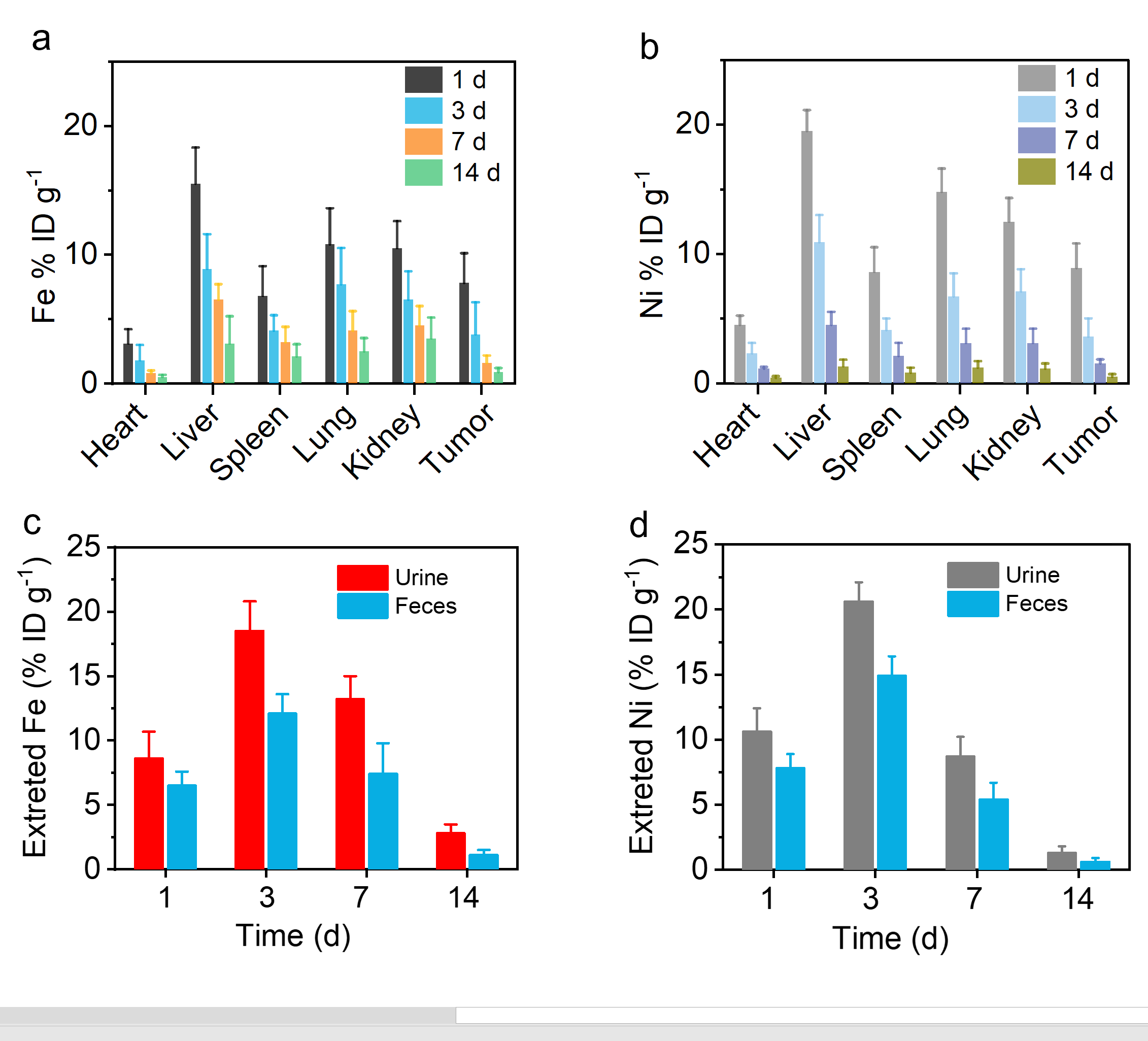


**Figure S26**. a) Biodistribution of Fe and b) Ni in the main organs and tumor and at different i.v. post-injection time points (1, 3, 6, and 14 days) (*n* = 3). c) Content of Fe and d) Ni in feces and urine at different time points.

Table S1 Summary of reported sonosensitizers for tumor therapy.

| Sonosensitizers | ROS species | Tumor model | Tumor inhibition rate (%) | Ref. |
| --- | --- | --- | --- | --- |
| Porphyrin-like  Carbon Nanosphere | ^1^O_2_, ·OH | 4T1 | 85 | [4] |
| TiO_2_-Ru Heterojunction | ^1^O_2_ | MB49 | 66.7 | [5] |
| C-ZnO | ^1^O_2_, ·OH | 4T1 | 90.4 | [6] |
| 2D Bi_2_MoO_6_ | ·O_2_^–^, ^1^O_2_,·OH | 4T1 | 96.6 | Ref.5a |
| 2D Fe-Bi_2_WO_4_ | ·O_2_^–^, ·OH | 4T1 | 68.4 | [7] |
| MnCO_3_ | ^1^O_2_, ·OH | 4T1 | 90.5 | [8] |
| Semiconducting Polymer | ^1^O_2_ | 4T1 | 89.6 | [9] |
| 2D WS_2_ | ^1^O_2_, ·OH | MCF-7 | -- | Ref.15a |
| 2D NiFe-LDH | ·O_2_^–^, ^1^O_2_,·OH | 4T1 | 91.7 | This work |

References:

[1] K. Fan, H. Chen, Y. Ji, H. Huang, P. M. Claesson, Q. Daniel, B. Philippe, H. Rensmo, F. Li, Y. Luo, L. Sun, *Nat. Commun.* **2016**, *7*, 11981.

[2] K. Galkowski, A. Mitioglu, A. Miyata, P. Plochocka, O. Portugall, G. E. Eperon, J. T.W. Wang, T. Stergiopoulos, S. D. Stranks, H. J. Snaith, R. J. Nicholas, *Energ. Environ. Sci*. **2016**, *9*, 962.

[3] M. Chen, M.G. Ju, H. F. Garces, A. D. Carl, L. K. Ono, Z. Hawash, Y. Zhang, T. Shen, Y. Qi, R. L. Grimm, D. Pacifici, X. C. Zeng, Y. Zhou, N. P. Padture, *Nat. Commun*. **2019**, *10*, 16.

[4] X. Pan, L. Bai, H. Wang, Q. Wu, H. Wang, S. Liu, B. Xu, X. Shi, H. Liu, *Adv. Mater.* **2018**, *30*, 1800180.

[5] G. Li, S. Wu, J. Liu, K. Wang, X. Chen, H. Liu, *Adv. Mater.* **2024**, *36*, 2401252.

[6] X. Pan, Z. Huang, J. Guo, Q. Wu, C. Wang, H. Zhang, J. Zhang, H. Liu, *Adv. Mater*. **2024**, DOI:[10.1002/adma.202400142](https://doi.org/10.1002/adma.202400142).

[7] Y. Ding, Y. Zhao, S. Yao, S. Wang, X. Wan, Q. Hu, L. Li, *Small* **2023**, *19*, 2300327.

[8] H. Zhang, X. Pan, Q. Wu, J. Guo, C. Wang, H. Liu, *Exploration* **2021**, *1*, 20210010.

[9] F. Wang, Y. Fan, Y. Liu, X. Lou, L. Sutrisno, S. Peng, J. Li, *Exploration* **2024**, [10.1002/EXP.20230100](https://doi.org/10.1002/EXP.20230100).
